# *Shigella* infections in the era of antimicrobial resistance: Insights into cephalosporin use

**DOI:** 10.1016/j.nmni.2026.101837

**Published:** 2026-09-01

**Authors:** Wenqing Wang, Yutian Qin, Yiqiong Wang, Xinyi Li, Dai Kuang, Mamy Jayne Nelly Rajaofera

**Affiliations:** NHC Key Laboratory of Tropical Disease Control, School of Life Sciences and Medical Technology, Hainan Medical University, Haikou, Hainan, 571199, China

**Keywords:** *Shigella*, Antimicrobial resistance, Cephalosporins, Extended-spectrum β-lactamases, Multidrug resistance, Vaccines, Phage therapy

## Abstract

**Background:**

*Shigella* causes severe bacterial diarrhea, especially in children in low- and middle-income countries. Rising antimicrobial resistance (AMR), including multidrug-resistant (MDR) and ESBL-producing strains, threatens empirical treatment, particularly cephalosporins.

**Methods:**

We conducted a structured narrative review of PubMed, Scopus, Web of Science, ScienceDirect, and WHO GLASS. Included studies comprised randomized trials, cohorts, case series, and relevant in vitro studies. Quality was appraised using Joanna Briggs Institute checklists; findings were synthesized narratively.

**Results:**

Third-generation cephalosporins (ceftriaxone, cefotaxime) remain highly effective for severe *Shigella* infections in hospitalized and pediatric patients when isolates are susceptible. Resistance varies regionally, with higher ESBL prevalence in East and South Asia. Resistance mechanisms include ESBLs, efflux pumps, PBP mutations, and altered membrane permeability. Susceptibility-guided therapy yields favorable outcomes, but ESBL/XDR strains cause treatment failures, longer hospital stays, and carbapenem use.

**Conclusion:**

Cephalosporins are critical for severe shigellosis but are increasingly compromised by AMR. Structured surveillance, susceptibility-guided therapy, and stewardship are essential. Complementary strategies (vaccines, probiotics, phage therapy, novel antibiotics) are urgently needed.

## Highlights

• Ceftriaxone and cefotaxime are key for severe, drug-resistant Shigella in children.
• ESBL production increasingly compromises cephalosporin efficacy in many regions.
• Resistance arises via enzymes, efflux pumps, PBP mutations, and mobile genetic elements.
• Future strategies include phage therapy, vaccines, and improved antimicrobial stewardship.

## 1 Introduction

*Shigella* is a genus of Gram-negative, facultatively anaerobic bacteria that stands as a leading cause of acute diarrheal disease globally, posing a substantial public health challenge [1,2]. The genus includes four major species including *Shigella dysenteriae*, *Shigella flexneri*, *Shigella boydii*, and *Shigella sonnei* [2,3], all of which are highly infectious and capable of causing shigellosis through a remarkably low infectious dose. Transmission occurs predominantly via the fecal-oral route through contaminated food, water, and direct person-to-person contact, making it particularly prevalent in areas with poor sanitation [4,5].

Children under five years of age bear the greatest burden, accounting for the majority of the estimated 164.7 million cases reported annually [6,7]. *Shigella* remains a leading cause of morbidity and mortality in low- and middle-income countries (LMICs) [4,8,9], with approximately 60,000 child deaths annually attributable to infection [1]. In LMICs, 2.0–7.0 per 100 children experience clinically significant *Shigella* infection during childhood, compared with 0.02 cases per child-year in high-income countries, underscoring the disproportionate burden in resource-limited settings [10].

Epidemiological patterns vary by region and over time. Historically, *S. sonnei* predominated in many LMICs; however, recent data indicate that *S. flexneri* has become the dominant species in these regions, while *S. sonnei* remains prevalent in high-income settings [4,6]. *S. dysenteriae* type 1 remains a significant cause of epidemic dysentery due to its Shiga toxin production [11]. Clinically, shigellosis presents with symptoms ranging from mild diarrhea to severe dysentery with bloody stools, fever, and abdominal pain [4,[11], [12], [13]]. Severe complications include hemolytic uremic syndrome, toxic megacolon, and septicemia [13,14].

The therapeutic landscape has been profoundly affected by the rapid emergence of AMR [15]. Misuse and overuse of antibiotics have facilitated the proliferation of resistant strains [16], with global estimates indicating that around 30% of antibiotic use is inappropriate, particularly in LMICs [17]. Resistance to traditional first-line antibiotics such as trimethoprim-sulfamethoxazole and ampicillin is widespread [18]. For instance, *Shigella* resistance to ampicillin reached 60% in the Tehran and Qazvin region [19], and 75% in China, while trimethoprim-sulfamethoxazole resistance reached 55% in China and up to 100% in Iran [19,20]. Resistance rates exceeding 50% have rendered these agents unreliable for empirical therapy. Resistance to fluoroquinolones and macrolides, including azithromycin, is also increasing [21,22]. Multidrug-resistant (MDR) *Shigella* strains, resistant to three or more antibiotic classes, are increasingly prevalent [23], and extensively drug-resistant (XDR) strains resistant to nearly all oral antibiotics pose a looming global threat [22,24].

Third-generation cephalosporins, including ceftriaxone and cefotaxime, have emerged as key alternatives, especially for severe infections in pediatric and hospitalized populations [25]. However, their effectiveness is threatened by rising resistance [21,[25], [26], [27], [28]], highlighting the need for updated evidence on clinical outcomes, regional susceptibility patterns, and strategies to guide antimicrobial stewardship. This review aims to critically synthesize current evidence on cephalosporin use for *Shigella* infections, assess regional AMR disparities, and identify knowledge gaps to inform future research, clinical practice, and public health interventions.

## 2 Materials and methods

### 2.1 Literature search strategy and study selection

This narrative review was conducted using a structured literature search in PubMed/MEDLINE, Scopus, Web of Science, ScienceDirect, and the WHO Global Antimicrobial Resistance and Use Surveillance System (GLASS) database. Search terms included “*Shigella*,” “shigellosis,” “bacillary dysentery,” “cephalosporin,” “ceftriaxone,” “cefotaxime,” “third-generation cephalosporin,” “antimicrobial resistance,” “AMR,” “ESBL,” “multidrug-resistant,” “MDR,” and “extensively drug-resistant,” “XDR.” The search covered the period from 1986 to 2025, as third-generation cephalosporins were introduced in the early 1980s and the earliest relevant clinical data on cephalosporin use in shigellosis began to appear from 1986 onwards. Reference lists of key articles and systematic reviews were manually screened to identify additional studies.

The initial database search yielded 4059 records. 1163 records were screened by title and abstract. Of these, 178 full-text articles were assessed for eligibility, and 105 studies met the inclusion criteria.

Inclusion and exclusion criteria were adapted post-search, based on the nature and scope of the retrieved literature, which is consistent with the flexible approach of a narrative review. Inclusion criteria encompassed original research studies (including clinical trials, observational studies, case series, and case reports) reporting clinical outcomes of Shigella infections treated with cephalosporins, providing antimicrobial susceptibility data, or describing resistance mechanisms in human populations, including pediatric and hospitalized patients. Systematic reviews and meta-analyses were also included for contextual background and cross-referencing of primary studies. Exclusion criteria included editorials, opinion pieces without primary data, narrative reviews that did not present original data or systematic methods, studies lacking clinical outcomes or susceptibility data, and non-human studies or in vitro studies without direct clinical relevance. Only English-language publications were included. Titles, abstracts, and full texts were independently screened by two reviewers, with disagreements resolved through discussion or consultation with a third reviewer.

### 2.2 Critical appraisal and evidence synthesis

All included studies underwent quality assessment using Joanna Briggs Institute checklists appropriate for their design. Studies were evaluated for methodological rigor, risk of bias, and internal validity, and classified as high, moderate, or low quality.

Data extraction captured study characteristics, antimicrobial susceptibility profiles, resistance mechanisms, clinical outcomes, and reported treatment failures. Extracted data were synthesized descriptively. Characteristics of included studies are summarized in Table S1. Resistance proportions, treatment success rates, and prevalence of ESBL- and XDR-producing strains were summarized. No formal meta-analysis was performed due to heterogeneity in study designs, populations, and outcome measures. Data were further analyzed by geographic region and *Shigella* species to identify epidemiological trends, highlight areas with high ESBL or XDR prevalence, and compare pediatric and general hospitalized populations. The narrative synthesis prioritized high-quality evidence while integrating observational and in vitro studies to contextualize resistance mechanisms.

## 3 Overview of *Shigella* infections

### 3.1 Pathogenesis and transmission

**Fig. 1:**
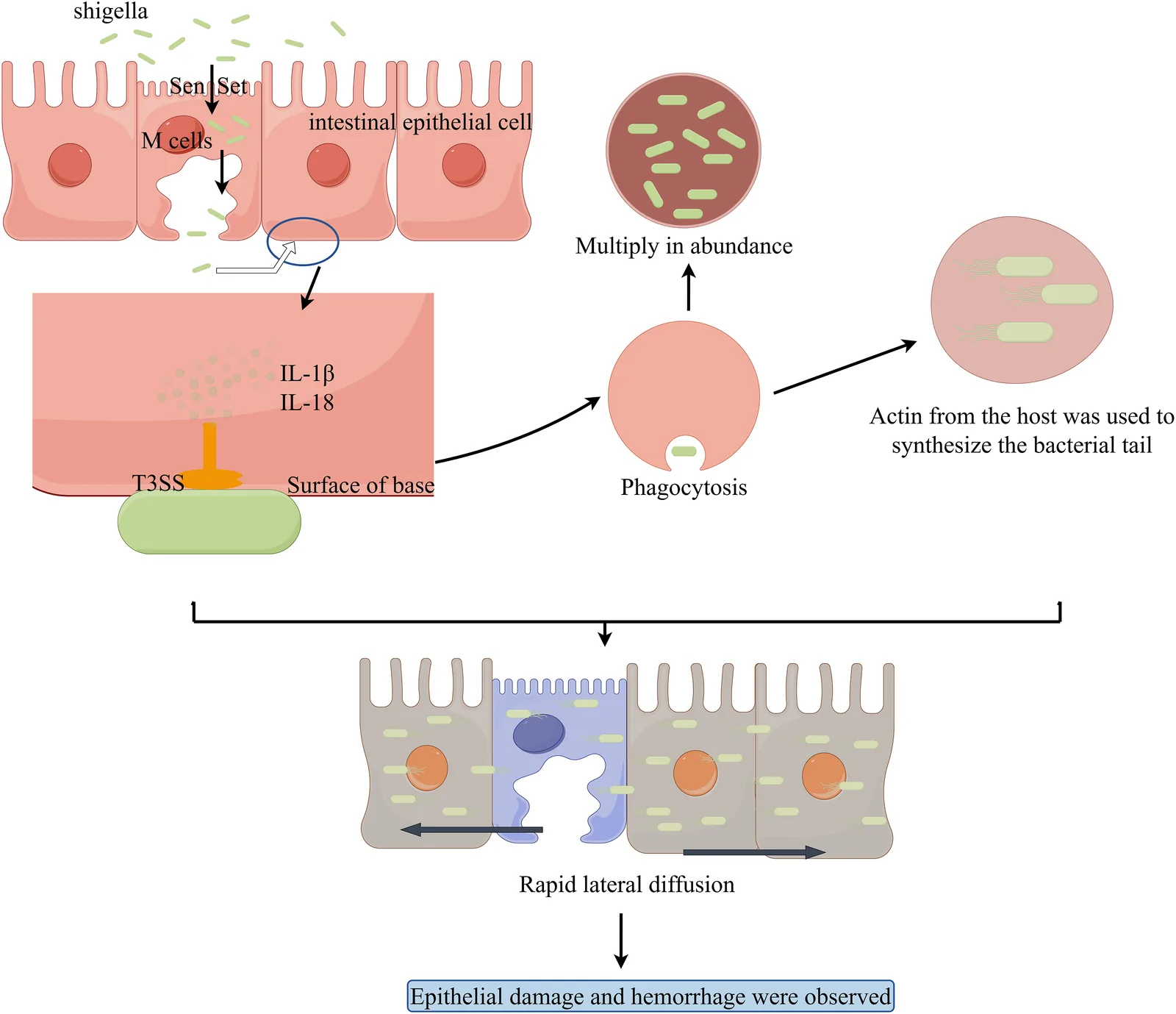
***Shigella* pathogenesis through M-cell mediated invasion and enterocyte colonization.** Set: *Shigella* Enterotoxin; Sen: *Shigella* Effector Protein M cells: Microfold cells. *Shigella* employs Sen and Set virulence factors to facilitate endocytosis by M cells and subsequent translocation across the intestinal epithelial barrier into the lamina propria. Following transcytosis, the bacteria target the basolateral surface of colonic epithelial cells, where the Type III Secretion System (T3SS) delivers specialized virulence factors that induce bacterial internalization. Once established intracellularly, *Shigella* undergoes rapid cytoplasmic replication and hijacks the host cell's actin polymerization machinery to generate propulsive actin tails, enabling directed motility and lateral spread to neighboring epithelial cells. This invasive process results in progressive tissue damage, inflammatory cell infiltration, and epithelial necrosis characteristic of shigellosis pathogenesis.

Shigellosis results from *Shigella* invasion of colonic epithelial cells, causing severe inflammation and tissue damage [4,29]. The bacterium utilized a Type III Secretion System (T3SS), encoded on the large virulence plasmid *pINV*, to inject effector proteins into host cells. Structurally, T3SS comprises a cytosolic sorting platform, a basal body spanning the inner and outer membranes, and an extracellular needle. Upon host cell contact, the needle tip senses the interaction and recruits the translocon proteins IpaB and IpaC, which are secreted to form a pore in the host membrane [30]. Through this pore, T3SS injects effectors that trigger cytoskeletal remodeling and facilitate M cell-mediated internalization. Following entry, the bacteria escape the phagosome, replicate intracellularly, and spread laterally via actin-based motility [31,32]. This process causes extensive epithelial injury (Fig. 1) manifesting as bloody diarrhea. *S. dysenteriae* type 1 produces Shiga toxin, potentially causing Hemolytic Uremic Syndrome (HUS) [33]. The inflammatory response contributes to abdominal pain, fever, and mucoid stools [34].

Transmission occurs via the fecal-oral route through contaminated water, food, or direct contact, particularly affecting regions with poor sanitation [35,36]. Children under five, immunocompromised individuals, and traveler are most susceptible [3]. A low infectious dose of 10-100 organisms enables rapid spread in epidemic settings [3,4,37]. Prevention relies on improved water access, sanitation, and hygiene education [38,39].

### 3.2 Current treatment guidelines

Management of shigellosis is guided by disease severity. Mild cases typically require only oral rehydration therapy, whereas moderate to severe infections necessitate antibiotics to shorten symptom duration, prevent complications, and limit transmission [18,25,40,41]. Choice of therapy depends on local resistance patterns, patient age, and comorbidities. Rising global AMR emphasizes the importance of evidence-based protocols and stewardship [18].

Fluoroquinolones, particularly ciprofloxacin, have historically been first-line therapy in adults due to rapid bactericidal activity and tissue penetration [42,43]. However, resistance has increased dramatically, particularly in South and Southeast Asia [[44], [45], [46]]. WHO 2025 reports indicate global ciprofloxacin resistance at 29.7% and levofloxacin at 32.7%, with Southeast Asia reaching 75.5% for ciprofloxacin [45]. A longitudinal study in Bangladesh confirmed this trend, rising from 0% in 2004 to 43% in 2012 [40]. Consequently, fluoroquinolone-resistant *Shigella* is now classified as high-priority on the WHO list (ranked 8th in 2024) [47].

Second-line options include azithromycin and third-generation cephalosporins (ceftriaxone, cefotaxime) [48,49]. Azithromycin is preferred for pediatric patients, pregnant women, and individuals with fluoroquinolone contraindications [50], b, but emerging resistance has been reported [44,51]. Cephalosporins remain essential for severe, hospitalized, or pediatric cases, particularly when fluoroquinolone or macrolide resistance is suspected [18]. However, ESBL-producing strains (e.g., CTX-M, TEM, SHV) hydrolyze third-generation cephalosporins, threatening their efficacy [52,53]. Lack of effective oral cephalosporins constrains outpatient therapy, especially in resource-limited settings [25].

Resistance impacts are evident even in high-income countries. For example, Israel reported ∼600 cases per 100,000 children aged 1-4 years [54]. In 2000, a 3-day intramuscular ceftriaxone regimen achieved 99% clinical cure in acute invasive diarrhea [55]. By 2026, only 36% of isolates remained susceptible to ceftriaxone, compared with 77% to azithromycin and 76% to ciprofloxacin [54]. These trends highlight the urgent need for antimicrobial stewardship, structured surveillance, and alternative strategies, including vaccines and phage therapy [47,54].

## 4 Antimicrobial resistance *in Shigella*

### 4.1 Cephalosporins and their mechanism of action

**Fig. 2:**
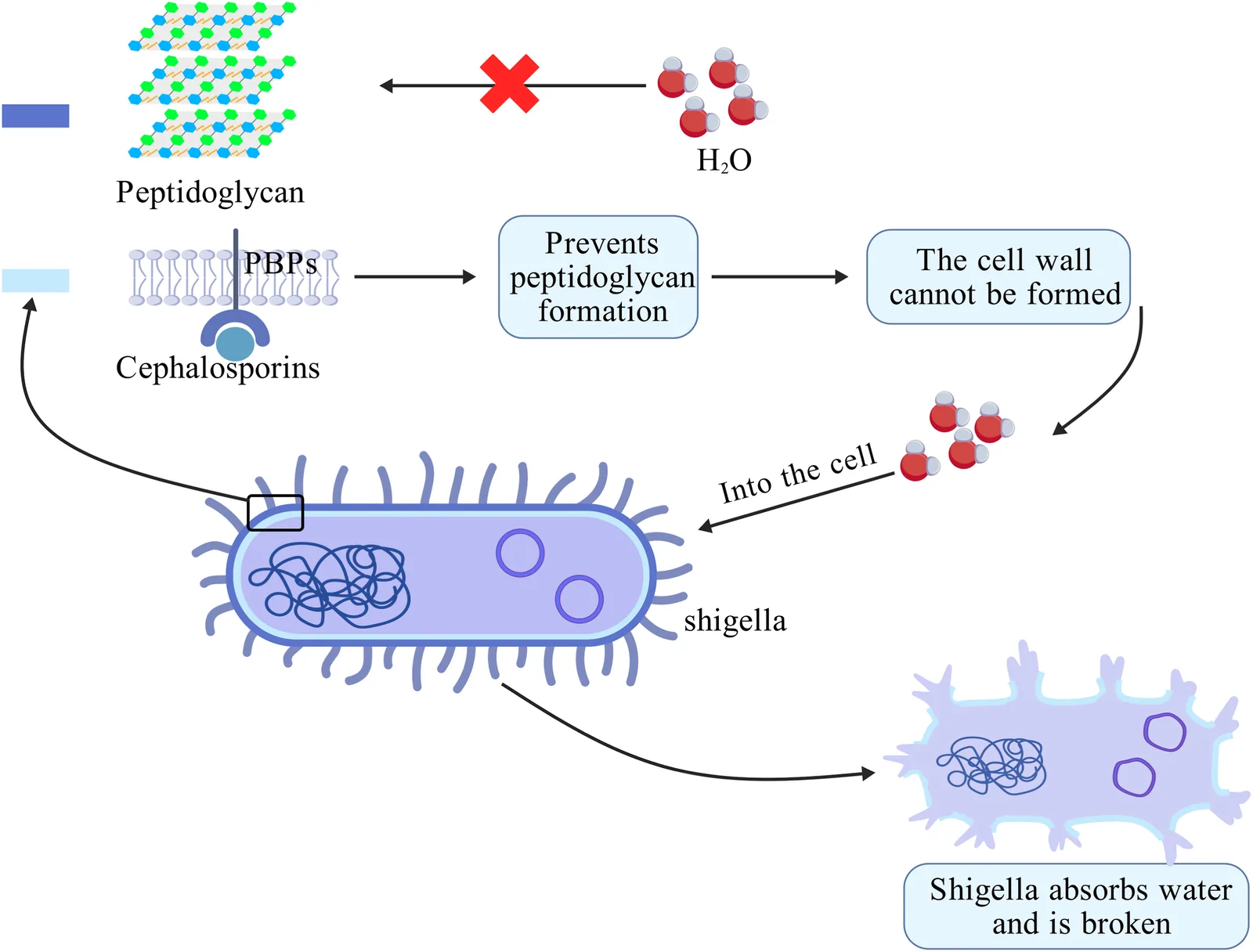
**Mechanism of cephalosporin-mediated growth inhibition in *Shigella*.** Cephalosporins (exemplified by cefotaxime) exert bactericidal activity by inhibiting peptidoglycan synthesis, the essential structural polymer that maintains bacterial cell wall integrity. The peptidoglycan layer provides critical osmotic protection by counteracting the influx of water molecules that would otherwise cause cellular swelling in hypotonic environments. When cephalosporins disrupt peptidoglycan cross-linking through inhibition of penicillin-binding proteins (PBPs), the compromised cell wall loses its structural integrity and osmotic resistance. Consequently, *Shigella* bacteria become vulnerable to osmotic stress and undergo cell lysis due to uncontrolled water influx and cytoplasmic swelling, resulting in bacterial death and therapeutic efficacy in the host environment.

Cephalosporins are β-lactam antibiotics that disrupt bacterial cell wall synthesis by binding to penicillin-binding proteins (PBPs), leading to bacterial cell lysis [56], Advanced generations show improved effectiveness against Gram-negative pathogens like *Shigella* through enhanced resistance to β-lactamase hydrolysis [21,57] (Fig. 2). Third generation cephalosporins such as ceftriaxone and cefotaxime are most important for treating shigellosis due to their strong Gram-negative activity.

The role of Cephalosporin in *Shigella* treatment has evolved with changing resistance patterns. Initially, trimethoprim sulfamethoxazole and ampicillin were commonly used, but rapid resistance emergence led to the use of third generation cephalosporins for severe cases [58,59]. Ceftriaxone and cefotaxime are now widely used in hospitalized patients, especially children, and preferred when fluoroquinolones or macrolides are not effective [25]. However, the rising prevalence of ESBL-producing *Shigella* strains presents a significant challenge. Globally, CTX-M type ESBL is the most common as shown in Table 1 [60]. These enzymes hydrolyze the β-lactam ring of cephalosporins, making the drugs ineffective and sometimes requiring alternative therapies such as carbapenems in severe cases [52]. (For detailed molecular regulation of *blaCTX-M* expression, see Section 4.3.1.)

**Table 1:** Extended spectrum beta lactamase types identified in *Shigella*, with corresponding cephalosporin resistance patterns, prevalence rates, and primary species.

Author	Antibiotic class(s)	Prevalence (%)	Primary species	Regions	Time
Liu et al., 2018 [1]	Cefotaxime	34.4	*S. flexneri*	Jiangsu, China	From 2013 to 2015
	Ceftazidime	24.1			
Li et al., 2015 [2]	Ciprofloxacin	10.3	*S. flexneri* *S. sonnei*	Shanghai, China	2007
	Trimethoprim/sulfamethoxazole	71.8			
Zhang et al., 2025 [3]	Ceftriaxone	30.49	*S. sonnei* *S. flexneri*	China	From 2004 to 2016
	Cefoperazone	27.11			
	Cefazolin	30.70			
Sabour et al., 2022 [4]	NF	NF	*S. flexneri* *S. dysenteriae* *S. sonnei*	Ardabil, northwest Iran	From January 2019 to December 2020
Khosravi et al., 2024 [5]	Ampicillin	88.2	*S. sonnei* *S. flexneri*	Northeastern Iran	From January 2017
	Cotrimoxazole	84.5			to December 2019
Aminshahidi et al., 2017 [6]	Ampicillin	95.1	*S. flexneri* *S. sonnei*	Shiraz, Iran	From August 2014
	Trimethoprim/Sulfamethoxazole	73.2			to February 2015
	Azithromycin	21.9			
	Ciprofloxacin	14.6			
Choia et al., 2023 [7]	Ceftriaxone	NF	*S. sonnei*	Temple, Texas,	January 2023
	Ciprofloxacin			USA	
Beeri et al., 2026 [8]	Ceftriaxone	64%	*S. sonnei*	Negev, southern	January 2020 to
	Azithromycin	23%		Israel	July 2024
	Ciprofloxacin	24%			
Shahi et al., 2026 [9]	Azithromycin	56.47%	*S. sonnei*	Ahvaz,	NF
		40.00%	*S. flexneri*	southwestern Iran	

1. Liu G, Qian H, Tang B, Chen Y, Kang H, Jiang F et al. Prevalence and characterisation of third-generation cephalosporin-resistant *Shigella flexneri* isolates from Jiangsu Province, China, 2013-2015. J Glob Antimicrob Resist 2018, 15:283-287. https://doi.org/10.1016/j.jgar.2018.08.012.

2. Li J, Li B, Ni Y, Sun J. Molecular characterization of the extended-spectrum beta-lactamase (ESBL)-producing *Shigella* spp. in Shanghai. Eur J Clin Microbiol Infect Dis 2015, 34(3):447-451. https://doi.org/10.1007/s10096-014-2244-2.

3. Zhang H, He S, Pang X, He X, Liu X, Qiu S et al. Regulatory role of *ISEcp1* and spacer sequences in *bla*(CTX-M)-mediated cephalosporin resistance in *Shigella*. J Glob Antimicrob Resist 2025, 44:127-134. https://doi.org/10.1016/j.jgar.2025.06.005.

4. Sabour S, Teimourpour A, Mohammadshahi J, Peeridogaheh H, Teimourpour R, Azimi T et al. Molecular detection and characterization of *Shigella* spp. harboring extended-spectrum beta-lactamase genes in children with diarrhea in northwest Iran. Mol Cell Pediatr 2022, 9(1):19. https://doi.org/10.1186/s40348-022-00152-0.

5. Khosravi M, Khosravi F, Pouresmaeil O, Aryan E, Meshkat Z, Safdari H et al. High prevalence of CTX-M-15 producing *Shigella* spp. isolated from patients with gastroenteritis in Northeast Iran. Acta Microbiol Immunol Hung 2024, 71(4):299-307. https://doi.org/10.1556/030.2024.02455.

6. Aminshahidi M, Arastehfar A, Pouladfar G, Arman E, Fani F. Diarrheagenic *Escherichia coli* and *Shigella* with High Rate of Extended-Spectrum Beta-Lactamase Production: Two Predominant Etiological Agents of Acute Diarrhea in Shiraz, Iran. Microb Drug Resist 2017, 23(8):1037-1044. https://doi.org/10.1089/mdr.2017.0204.

7. Choia H, Navarathnaa DH, Harstona BL, Hwanga M, Coronaa B, San Juana MR et al. Case of Extensively Drug-Resistant *Shigella sonnei* Infection, United States. Emerg Infect Dis 2023, 29(8). https://doi.org/10.3201/eid2908.230411.

8. Beeri Y, Ben Shitrit I, Hazan G, Beniacar H, Sagi O, Soyonov E et al. *Shigella* PCR-positive Gastroenteritis in Children: Insights From a Comparison Between Culture-positive and Culture-negative Cases. Pediatr Infect Dis J 2026, 45(1):61-67. https://doi.org/10.1097/INF.0000000000004965.

9. Shahi F, Abbasi Montazeri E, Khaghani S, Moradi M, Ahmad Khosravi N, Saki M. Investigation of the azithromycin resistance rate and related genes involved in this resistance in clinical *Shigella* species collected from children with diarrhea in Ahvaz, Southwest Iran. Mol Biol Rep 2026, 53(1):353. https://doi.org/10.1007/s11033-025-11420-3.

Although cephalosporins remain essential for severe shigellosis treatment, their effectiveness is threatened by resistance, particularly in areas with high ESBL prevalence [60]. The limited availability of oral formulations creates challenges for outpatient management [25]. Therefore, cephalosporin use should be guided by susceptibility testing and integrated into antimicrobial stewardship programs to preserve their clinical utility.

### 4.2 Antimicrobial resistance trends and dissemination pathways

Antimicrobial resistance in *Shigella* represents a growing public health crisis. Historically effective first line antibiotics such as trimethoprim sulfamethoxazole and ampicillin became widely ineffective by the late 20th century [61], leading to the use of fluoroquinolones and macrolides. In recent decades, multidrug resistant strains have emerged rapidly, particularly in South Asia, sub Saharan Africa, and Latin America [62]. Current surveillance reveals alarming trends. In some regions, fluoroquinolone resistance exceeds 90% [6], azithromycin resistance surpasses 60% in endemic areas [63], and ESBL-producing *Shigella* threatens third-generation cephalosporins [21,25]. Regional differences emphasize the need for localized surveillance. South Asia reports high fluoroquinolone resistance, whereas parts of Africa show lower rates [61,64]. In Africa, the pooled prevalence of ESBL and carbapenem-resistant *Shigella* is estimated at 41.2%, with *S. flexneri* being the most prevalent species (34.5%). The most common ESBL genes are *blaTEM* (25.9%), *blaOXA-1* (25.7%), and *blaCTX-M* (10.8%), while carbapenemase genes such as *blaNDM*, *blaKPC*, and *blaIMP* are rare (<1.5%) [65]. In one Chinese study of *S. flexneri*, CTX-M-1 group enzymes accounted for 43.8%, CTX-M-9 group for 42.5%, TEM-1 and TEM-1b for 34.2%, and SHV-12 for 1.4% of ESBL isolates [66].

These geographic disparities are driven, in part, by the rapid dissemination of plasmid-mediated resistance mechanisms [67]. Plasmids carry multiple resistance genes and facilitate horizontal gene transfer through conjugation, transformation, and transduction [68,69]. In *Shigella*, plasmid-mediated resistance genes encode β-lactamases, quinolone resistance determinants [46], macrolide resistance genes [70], and aminoglycosides, resulting in multidrug-resistant phenotypes [21,71]. Global dissemination of plasmids carrying *blaCTX-M* genes has been extensively documented in isolates from South Asia, sub-Saharan Africa, and the Middle East [72]. In South African *Shigella* strains, plasmids with replicon types such as IncFIA and IncFIB are commonly associated with the carriage of resistance genes like *tet(A)* and *floR*, highlighting the role of conjugative plasmids in regional resistance spread [73]. The spread of plasmid-mediated resistance is exacerbated by antibiotic misuse [21,69], poor sanitation [4,74], and global travel patterns [72]. Addressing the crisis requires comprehensive surveillance [12], antimicrobial stewardship, and novel therapeutic strategies [21].

### 4.3 Mechanisms of drug resistance

#### 4.3.1 Enzymatic resistance through extended-spectrum β-lactamases

Plasmid-mediated ESBLs (such as CTX-M, TEM) hydrolyze the beta-lactam ring, preventing antibiotic binding to target PBPs and thereby inhibiting bacterial cell wall synthesis [21]. The emergence of ESBL-producing *Shigella* strains has significantly compromised the effectiveness of cephalosporins [75] and limited their use in heavily managed or MDR infections [25]. Common ESBL types, including CTX-M, TEM, and SHV variants, are frequently identified in *S. flexneri* and *S. sonnei* isolates [69], with higher prevalence observed in areas characterized by extensive antibiotic use and inadequate surveillance systems [46,60]. The insertion sequence *ISEcp1*, frequently located upstream of *blaCTX-M*, acts as a strong promoter and enables high-level expression of the resistance gene. Spacer sequences between *ISEcp1* and *blaCTX-M* (e.g., 42, 48, 127 bp) further fine-tune expression levels, contributing to varied resistance phenotypes among clinical isolates [76]. Table 2 details on common drug resistance patterns, corresponding genetic determinants, primary circulating species, and prevalence rates [66,[76], [77], [78], [79], [80], [81]].

**Table 2:** Resistance mechanisms in *Shigella*, their associated resistance genes, and the species in which they have been reported.

Author	Resistance gene	Carriage rate (%)	Resistance mechanism	Primary species
Liu et al., 2018 [1]	*blaTEM*	16.4	Extended-spectrum β-lactamases (ESBLs)	*S. flexneri*
	*blaCTX-M-1*	32.9		
	*blaCTX-M-9*	28.8		
Li et al., 2015 [2]	CTX-M-14	69.2	Extended-spectrum β-lactamases (ESBLs)	*S. flexneri* *S. sonnei*
	CTX-M-15	15.4		
	CTX-M-3	10.3		
	CTX-M-55	5.1		
Zhang et al., 2025 [3]	IS*Ecp1*	90.09	Extended-spectrum β-lactamases (ESBLs)	*S. sonnei* *S. flexneri*
	IS*Ecp1-CTX-M*	67.19		
Sabour et al., 2022 [4]	*blaCTX*	65.3	Extended-spectrum β-lactamases (ESBLs)	*S. flexneri* *S. dysenteriae* *S. sonnei*
	*blaTEM*	61.2		
Khosravi et al., 2024 [5]	*sen*	72.7	Extended-spectrum β-lactamases (ESBLs)	*S. sonnei* *S. flexneri*
	*blaCTX-M-15*	100		
	*blaTEM*	100		
Aminshahidi et al., 2017 [6]	CTX	53.7	Extended-spectrum β-lactamases (ESBLs)	*S. flexeneri* *S. sonnei*
	SXT	73.2		
	AMP	95.1		
	CTR	56.1		
Choia et al., 2023 [7]	*blaCTX-M-27*(Putative**)**	NF	NF	*S. sonnei*
	qnrB19, *gyrA* (p.S83L) (Putative**)**			

NF: *not found*.

References.

1. Liu G, Qian H, Tang B, Chen Y, Kang H, Jiang F et al. Prevalence and characterisation of third-generation cephalosporin-resistant *Shigella flexneri* isolates from Jiangsu Province, China, 2013-2015. J Glob Antimicrob Resist 2018, 15:283-287. https://doi.org/10.1016/j.jgar.2018.08.012.

2. Li J, Li B, Ni Y, Sun J. Molecular characterization of the extended-spectrum beta-lactamase (ESBL)-producing *Shigella* spp. in Shanghai. Eur J Clin Microbiol Infect Dis 2015, 34(3):447-451. https://doi.org/10.1007/s10096-014-2244-2.

3. Zhang H, He S, Pang X, He X, Liu X, Qiu S et al. Regulatory role of *ISEcp1* and spacer sequences in *bla*(CTX-M)-mediated cephalosporin resistance in *Shigella*. J Glob Antimicrob Resist 2025, 44:127-134. https://doi.org/10.1016/j.jgar.2025.06.005.

4. Sabour S, Teimourpour A, Mohammadshahi J, Peeridogaheh H, Teimourpour R, Azimi T et al. Molecular detection and characterization of *Shigella* spp. harboring extended-spectrum beta-lactamase genes in children with diarrhea in northwest Iran. Mol Cell Pediatr 2022, 9(1):19. https://doi.org/10.1186/s40348-022-00152-0.

5. Khosravi M, Khosravi F, Pouresmaeil O, Aryan E, Meshkat Z, Safdari H et al. High prevalence of CTX-M-15 producing *Shigella* spp. isolated from patients with gastroenteritis in Northeast Iran. Acta Microbiol Immunol Hung 2024, 71(4):299-307. https://doi.org/10.1556/030.2024.02455.

6. Aminshahidi M, Arastehfar A, Pouladfar G, Arman E, Fani F. Diarrheagenic *Escherichia coli* and *Shigella* with High Rate of Extended-Spectrum Beta-Lactamase Production: Two Predominant Etiological Agents of Acute Diarrhea in Shiraz, Iran. Microb Drug Resist 2017, 23(8):1037-1044. https://doi.org/10.1089/mdr.2017.0204.

7. Choia H, Navarathnaa DH, Harstona BL, Hwanga M, Coronaa B, San Juana MR et al. Case of Extensively Drug-Resistant *Shigella sonnei* Infection, United States. Emerg Infect Dis 2023, 29(8). https://doi.org/10.3201/eid2908.230411.

#### 4.3.2 Efflux pump systems

Efflux pumps are membrane-embedded protein complexes that actively expel antimicrobial agents from bacterial cells, thereby lowering intracellular drug concentrations and reducing antibacterial activity [82]. Six major efflux pump families have been described in Gram-negative bacteria: the ATP-binding cassette (ABC) superfamily, the major facilitator superfamily (MFS), the resistance–nodulation–division (RND) family, the multidrug and toxic compound extrusion (MATE) family, the small multidrug resistance (SMR) family, and the Proteobacterial antimicrobial compound efflux (PACE) family [74,83]. In *Shigella*, the AcrAB-TolC system contributes to multidrug resistance by reducing intracellular concentrations of β-lactams, particularly when combined with outer-membrane permeability defects [84,85]. In *S. flexneri*, AcrAB-TolC also promotes epithelial cell invasion [85]. Members of the SMR family, including YnfA and EmrE, function as H^+^/drug antiporters; YnfA has been functionally confirmed as a multidrug efflux transporter in *Shigella* [83]. Genomic surveillance shows that efflux determinants are widespread. In South African *S. flexneri* strains, the multidrug efflux gene *mdfA* is present in nearly all sequenced genomes [73]. Together, efflux pump systems limit antibiotic accumulation and contribute to cephalosporin resistance [82,83].

#### 4.3.3 Penicillin-binding protein (PBP) mutations

PBP mutations involve chromosomal alterations in the genes encoding these essential enzymes responsible for bacterial cell wall synthesis in *Shigella*. These genetic modifications alter the 3D structure of PBPs and reduce binding affinity, thereby preventing β-lactam antibiotics from efficiently binding to their intended molecular targets [86]. Structural changes in PBPs reduce the formation of stable antibiotic-enzyme complexes, thereby impairing the inhibition of peptidoglycan cross-linking processes essential for cell wall integrity. As result, these mutations lead to loss of bacterial growth inhibition and drive the development of the β-lactam resistance pattern observed in clinical *Shigella* isolates [46].

#### 4.3.4 Altered cell membrane permeability

**Fig. 3:**
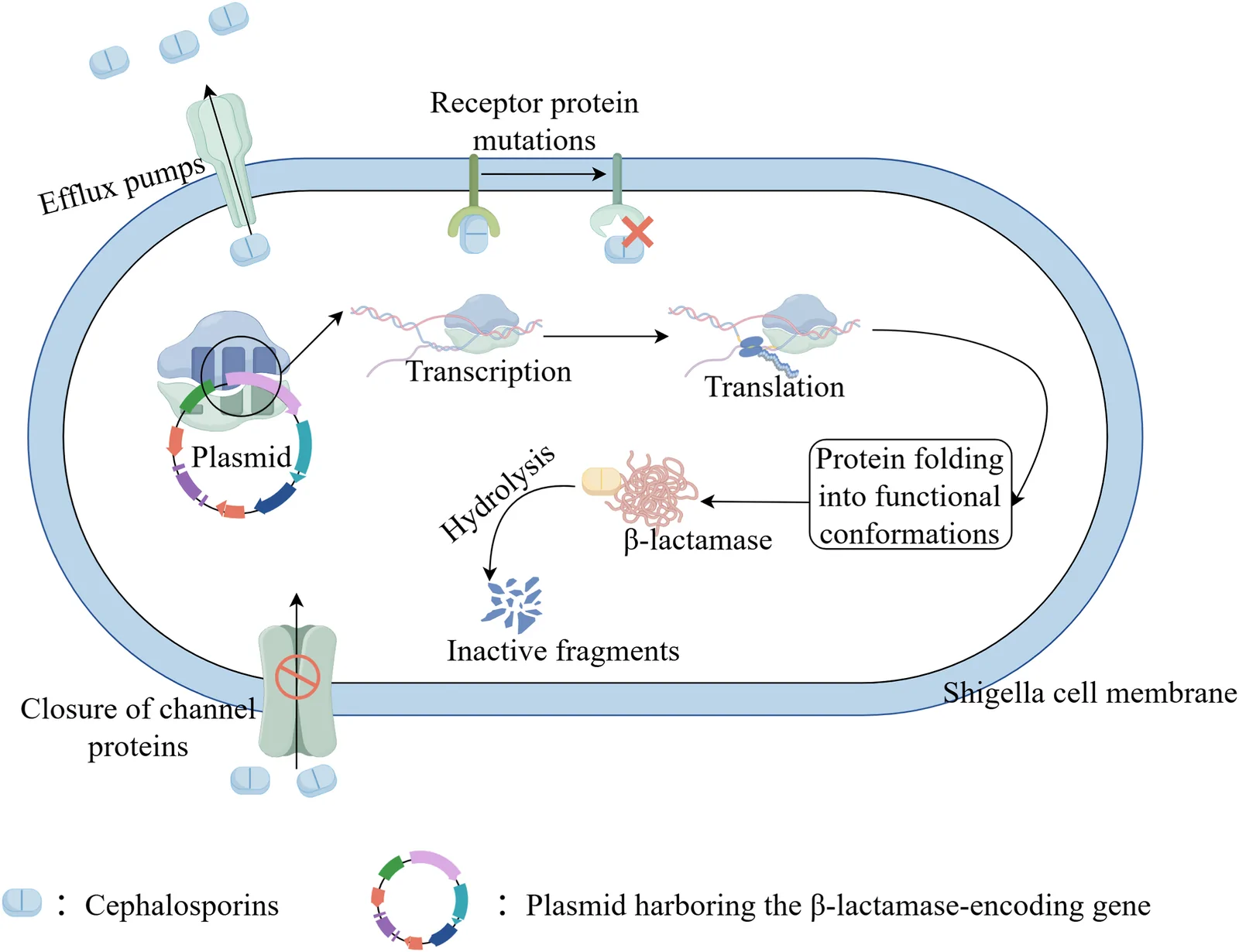
**Multifaceted mechanisms of cephalosporin resistance in *Shigella*.** This diagram illustrates four distinct resistance mechanisms employed by *Shigella* to counteract cephalosporin activity: β-lactamase production, altered membrane permeability, penicillin-binding protein mutations, and enhanced efflux pump activity. β-lactamases enzymatically hydrolyze the β-lactam ring structure of cephalosporins, rendering them inactive through cleavage into non-functional fragments. Downregulation of outer membrane porins (*OmpF*/*OmpC*) reduces antibiotic permeability, leading to extracellular drug accumulation and insufficient intracellular concentrations. Structural mutations in penicillin-binding proteins diminish antibiotic binding affinity, compromising the inhibition of peptidoglycan synthesis and cell wall formation. Enhanced efflux pump systems actively transport cephalosporins out of the bacterial cell, maintaining subtherapeutic intracellular drug concentrations and preventing effective antimicrobial action. These complementary resistance mechanisms can operate simultaneously, creating multidrug-resistant phenotypes that significantly challenge therapeutic interventions.

Altered cell membrane permeability constitutes a significant resistance mechanism through which *Shigella* modifies antibiotic accessibility by reducing the expression of essential outer membrane porins, particularly OmpF and OmpC [87,88]. These porins facilitate passive diffusion of antibiotics. By reducing porin expression, *Shigella* diminishes membrane permeability, restricts antibiotic penetration (Fig. 3), and maintains subtherapeutic intracellular concentrations, ultimately contributing to treatment failure.

### 4.4 Regional comparative synthesis of antimicrobial resistance

Substantial geographic heterogeneity in *Shigella* AMR patterns exists, yet direct comparison across regions is constrained by uneven data availability. In East Asia, data from China indicate well-established resistance to third-generation cephalosporins. In Jiangsu Province (2013–2015), cefotaxime resistance in *S. flexneri* was 34.4% and ceftazidime resistance 24.1% [66]. Li et al. reported that 18.1% of *Shigella* isolates in Shanghai (2007) produced ESBLs; among these ESBL-producers, ciprofloxacin resistance was 10.3% and trimethoprim-sulfamethoxazole resistance was 71.8% [77]. A long-term Chinese study (2004–2016) analyzing 1393 cephalosporin-resistant *Shigella* strains reported *ISEcp1* and *ISEcp1-CTX-M* element carriage rates of 90.09% and 67.19%, respectively [76]. These findings suggest that although fluoroquinolone resistance was historically lower in East Asia than in South Asia, cephalosporin resistance is now well entrenched.

In the Middle East, multiple studies from Iran show very high resistance to older antibiotics, with more variable resistance to cephalosporins and fluoroquinolones. In northwest Iran (2019–2020), Sabour et al. reported that although 65.3% of *Shigella* isolates carried *blaCTX-M* and 61.2% carried *blaTEM*, phenotypic ESBL production was only 10.2%; specific cephalosporin resistance rates were not reported [80]. In northeastern Iran (2017–2019), ampicillin resistance reached 88.2% and cotrimoxazole 84.5% [79]. In Shiraz (2014–2015), ampicillin resistance was 95.1% and trimethoprim-sulfamethoxazole 73.2%, while ciprofloxacin resistance was only 14.6% and azithromycin 21.9% [81]. A recent study from southwestern Iran (2026) found that among azithromycin-resistant *Shigella* isolates, 56.5% were *S. sonnei* and 40.0% were *S. flexneri*, with overall azithromycin resistance of 67.5% [51]. Thus, in Iran, fluoroquinolone and cephalosporin susceptibility remain partially preserved in some regions, but resistance to macrolides and older agents is alarmingly high.

In high-income countries (HICs) such as Israel and the USA, the picture is different. In southern Israel (2020–2024), ceftriaxone resistance reached 64%, ciprofloxacin resistance 24%, and azithromycin resistance 23% [54]. This represents a dramatic erosion of cephalosporin efficacy even in a high-resource setting that historically enjoyed high cure rates with ceftriaxone [55]. In the USA, an extensively drug-resistant *S. sonnei* case from Texas (2023) showed resistance to both ceftriaxone and ciprofloxacin [78]. Canadian data have documented ceftriaxone-resistant *S. sonnei* clusters linked to international travel [43]. HICs therefore face imported and locally transmitted resistant strains, but they benefit from better genomic surveillance and greater access to second-line agents such as carbapenems.

Limited peer-reviewed studies from South Asia reporting comparable cephalosporin resistance metrics, and from Africa only a meta-analysis of ESBL prevalence (41.2%) without study-level detail for direct comparison [65]. Consequently, a robust three-region (South Asia, Africa, HICs) comparative synthesis is not possible from the retrieved literature. This gap underscores an urgent need for standardized surveillance in under-represented regions. Overall, available data indicate that cephalosporin resistance in *Shigella* is widespread across East Asia, the Middle East, and HICs, though prevalence varies. South Asia is known from other reports to have the highest fluoroquinolone resistance globally, but direct inclusion in this synthesis was not possible due to insufficient data meeting our inclusion criteria. Future research should prioritize harmonized susceptibility testing and reporting from South Asia and Africa to enable true global comparisons.

## 5 Efficacy of cephalosporins in *Shigella* infections

Clinical studies included in this review were categorized according to the JBI hierarchy of evidence. Randomized controlled trials and large cohort studies were considered high quality evidence, whereas smaller cohort studies and outbreak investigations were classified as moderate quality evidence. Case reports and small case series were regarded as low quality evidence and were included primarily for illustrative purposes. Because this study was conducted as a narrative review rather than a formal systematic review, no quantitative risk of bias assessment or meta-analysis was performed.

### 5.1 Clinical studies and evidence

Historically, cephalosporins were secondary to trimethoprim-sulfamethoxazole and ampicillin, but rapid resistance to those agents has elevated third-generation cephalosporins to important therapeutic options for severe *Shigella* infections [58,59]. Third-generation cephalosporins, particularly ceftriaxone and cefotaxime, have emerged as a mainstay of therapy for severe and MDR *Shigella* infections, especially in pediatric populations. [25,43]. Multicenter studies conducted in South Asia and Africa, classified as high quality evidence, demonstrated ceftriaxone's superior efficacy in severe dysentery caused by multidrug resistant *Shigella* strains, with high clinical cure rates, shorter hospitalization duration, and reduced mortality compared with conventional treatments [89]. Cefotaxime has proven particularly valuable for systemic infections, including *Shigella* bacteraemia, and remains effective in regions with high fluoroquinolone and azithromycin resistance rates [90]. However, ESBL-mediated resistance (Section 4.3.1) compromises cephalosporin utility, making pre-treatment susceptibility testing essential [91]. Emergence of ESBL resistance can occur within days during hospital treatment, so repeat testing may be required if rapid clinical improvement is not observed.

### 5.2 Clinical evidence and treatment outcomes

#### 5.2.1 Successful outcomes

A randomized controlled trial in Bangladesh demonstrated complete clinical cure in 20 children with severe dysentery caused by ciprofloxacin-resistant *S. flexneri* using intravenous ceftriaxone (50 mg/kg/day for 5 days) [92]. All patients achieved symptom resolution within 48-72 h, with negative stool cultures by day 3 and no relapses during follow-up, supporting WHO guidelines for empirical therapy [22]. Additional form low quality case reports from India documented successful treatment of resistant *S. flexneri* with cefoperazone-sulbactam [58], while 2024 data from British Columbia confirmed ceftriaxone susceptibility in dominant MDR *S. sonnei* clones [93]. During a 2019 Belgian outbreak involving 163 *S. sonnei* cases linked to Central American travel, intravenous cefotaxime achieved 90% clinical cure rates in severely affected children, despite strain resistance to ciprofloxacin and azithromycin [94]. Conversely, a 2005 Taiwan outbreak revealed ceftriaxone-resistant *S. sonnei* producing CMY-2 β-lactamase, highlighting the critical importance of susceptibility testing [95].

#### 5.2.2 Challenges and failures

A 2017 case from Nepal involved a 5-year-old child with bacteremia caused by CTX-M-15 ESBL-producing *S. flexneri* presenting with septic shock and disseminated intravascular coagulation [96]. Empirical ceftriaxone failed, but meropenem (10 mg/kg every 8 h) achieved clinical improvement within 48 h, reflecting regional ESBL prevalence driven by plasmid-mediated *blaCTX-M* genes. This case demonstrates severe systemic complications from ESBL-producing *Shigella*, particularly in pediatric populations. In 2023, a 33-year-old immunocompromised traveler from Southeast Asia with XDR *S. sonnei* (sequence type 152) harboring *blaCTX-M-27* and *gyrA* mutations was described [78]. Despite combination therapy with aztreonam and tigecycline, clinical response was delayed and symptoms persisted for 14 days. This case highlights the growing challenge of multidrug resistant *Shigella* in international travelers, where ESBL production combined with fluoroquinolone resistance severely limits therapeutic options.

Third-generation cephalosporins remain cornerstone therapy for severe *Shigella* infections, particularly in pediatric and hospitalized populations. However, escalating ESBL-mediated resistance increasingly compromises their efficacy, forcing reliance on carbapenems and raising treatment failure risks. Addressing these challenges requires coordinated global efforts encompassing enhanced resistance surveillance, antimicrobial stewardship, and development of novel therapeutics and vaccines [97,98]. Without such interventions, rising resistance threatens to render current treatments obsolete, substantially increasing the public health burden of shigellosis.

## 6 Knowledge gaps, limitations, and forward-looking priorities

Current evidence indicates that third-generation cephalosporins (ceftriaxone, cefotaxime) remain critical for severe *Shigella* infections, particularly in pediatric and hospitalized populations. Clinical trials and multicenter studies report high cure rates and reduced hospitalization duration [89,90], while case reports illustrate challenges posed by ESBL-producing and XDR strains [78,96]. Despite these findings, several critical knowledge gaps persist. High-quality prospective trials, particularly randomized controlled trials comparing cephalosporins with alternative agents in MDR *Shigella*, are scarce. Geographic coverage is uneven, with most studies concentrate in South Asia and Africa, leaving resistance trends in other regions under-characterized. The relationship between molecular resistance mechanisms (ESBL genes, PBP mutations, efflux pumps) and clinical outcomes is not fully understood. Vulnerable populations (pediatric, immunocompromised, travelers) are underrepresented, and evidence for outpatient oral cephalosporin use remains minimal.

Limitations of the current literature include heterogeneity in study designs, populations, outcome measures, and reporting standards, which precludes quantitative synthesis. Most studies are observational or case-based, representing low-quality evidence, and resistance patterns evolve rapidly; older data (e.g., pre-2010) may not accurately reflect current epidemiology. These factors limit generalizability and underscore the need for prospective studies, standardized surveillance, and well-designed clinical trials.

Forward-looking recommendations are directly linked to these gaps. The escalating AMR in *Shigella* infections necessitates urgent development of innovative therapeutic strategies and enhanced surveillance systems. Next-generation antibiotics targeting resistant *Shigella* strains are a critical research priority. β-lactamase inhibitors that restore cephalosporin efficacy [99] and broad-spectrum siderophore-conjugated antibiotics that exploit bacterial iron uptake pathways [100], offer promising avenues for overcoming MDR and XDR *Shigella* [101]. Non-antibiotic strategies are emerging as valuable complementary approaches. Probiotics, particularly *Lactobacillus* and *Bifidobacterium* strains, have demonstrated efficacy in clinical trials by restoring gut microbiota balance and inhibiting pathogen colonization. Vaccine development, including live-attenuated, conjugate, and subunit candidates [8,98,102], aims to provide long-lasting immunity while reducing antibiotic dependence. Phage therapy offers a precision approach using bacteriophages to selectively target resistant bacteria [100,103], sparing beneficial microbiota and minimizing selective pressure for resistance [104,105]. Enhanced surveillance systems and robust antimicrobial stewardship programs are essential to monitor resistance trends, guide empiric therapy, and preserve the efficacy of cephalosporins and other critical antibiotics.

Conceptually, cephalosporin efficacy is influenced by pathogen resistance mechanisms, patient characteristics, and treatment context (hospitalized vs. outpatient). Recognizing these relationships clarifies where evidence is insufficient and informs future research, clinical decision-making, and public health strategies. A coordinated global effort combining novel therapeutics, enhanced surveillance, rigorous stewardship, and vaccine deployment is essential to overcome the challenges posed by AMR in *Shigella* and ensure effective, sustainable management of shigellosis.

## 7 Conclusion

Future strategies for managing *Shigella* infections should integrate novel therapeutic approaches with strengthened antimicrobial stewardship and comprehensive surveillance systems. The development of new antimicrobials, vaccines, probiotics, and phage therapy offers potential solutions to current treatment limitations. Concurrently, robust stewardship programs and enhanced surveillance are essential to preserve the efficacy of existing antibiotics and limit the spread of resistant strains. A coordinated, global effort that combines these measures is critical to addressing AMR in *Shigella* and ensuring sustainable, effective management of shigellosis.

## CRediT authorship contribution statement

**Wenqing Wang:** Writing – original draft. **Yutian Qin:** Data curation. **Yiqiong Wang:** Data curation. **Xinyi Li:** Data curation. **Dai Kuang:** Funding acquisition. **Mamy Jayne Nelly Rajaofera:** Writing – review & editing, Visualization, Validation, Supervision.

## Data availability statement

This article is a narrative review and does not include any original datasets. All data generated or analyzed during this study are included in this published article and its supplementary information files.

## Ethical approval

Not required.

## Generative AI statement

Generative AI tools were used only to assist with language polishing and improving clarity of expression. No AI tools were used to generate, analyze, or interpret scientific content. All scientific judgments, conclusions, and revisions were made by the authors.

## Declaration of competing interest

The authors declare the following financial interests/personal relationships which may be considered as potential competing interests: Dai Kuang reports financial support was provided by Hainan Medical University. Dai Kuang reports a relationship with Hainan Provincial Natural Science Foundation of China that includes: funding grants 825RC764. If there are other authors, they declare that they have no known competing financial interests or personal relationships that could have appeared to influence the work reported in this paper.
