# Supplementary material for: Shigella infections in the era of antimicrobial resistance: Insights into cephalosporin use
Source: New Microbes New Infect. 2026 Sep 1;73:101837. doi: 10.1016/j.nmni.2026.101837 (PMC13559839; doi:10.1016/j.nmni.2026.101837)
Supplement: Supplementary file 1 [file mmc1.docx]

**Table S1.** Characteristics of study included in this review.

| **Study** | **Country/Time** | **Study Design** | **Sample Size**  **Population** | **Key Outcomes** | | **Quality**  **Rating** |
| --- | --- | --- | --- | --- | --- | --- |
| Shahiet al. 2026 [1] | Iran, 2026 | Observational study | 126  children | Azithromycin resistance 67.5% (85/126); 98.8% *mphA*, 8.2% *ermB*; efflux in 9.4%. MDR 77.0%, XDR 17.5%. High resistance: nalidixic acid (99.2%), ampicillin (91.3%), cefazolin (84.1%), TMP‑SMX (73.0%). Most effective: imipenem (96.8% susceptible), chloramphenicol (90.5%), levofloxacin/amikacin/meropenem (88.9%). | | Moderate quality |
| WHO 2025 [2] | Global, 2025 (data 2023) | Observational study | 6,318 *Shigella* spp. isolates from gastrointestinal infections reported by 20 countries | Global resistance: ciprofloxacin 29.7% (22.9–37.5), azithromycin 25.6% (14.2–41.5), ceftriaxone 27.8% (19.1–38.5). Highest ciprofloxacin resistance in South East Asia (75.5%, 58.1–87.3); highest azithromycin in South East Asia (45.3%, 22.3–70.4) and Western Pacific (44.6%, 36.3–53.3). No significant global trend for ciprofloxacin resistance (2018–2023). | | Moderate quality |
| Sati et al. 2025 [3] | International, 2024 | *In vitro* study | 24 resistant bacterial pathogens, including fluoroquinolone-resistant *Shigella* | Fluoroquinolone-resistant *Shigella* spp. scored 70% (ranked among top community-acquired pathogens) and classified as high priority in the 2024 WHO BPPL. | | Moderate quality |
| Somda et al. 2025 [4] | Africa, 1999–2024 | Review | 18 studies, 11 African countries, 403 ESBL/carbapenemase-producing *Shigella* isolates | Pooled prevalence of ESBL/CR *Shigella*: 41.2% (95% CI: 22.8–62.4); *S. flexneri* most prevalent (34.5%); commonest genes: *blaTEM* (25.9%), *blaOXA-1* (25.7%), *blaCTX-M* (10.8%); carbapenemase genes rare (<1%).  Moderate quality | | Moderate quality |
| Zhang et al. 2025 [6] | China, 2004–2016 | Randomized trials | 1,393 cephalosporin-resistant *Shigella* (778 *S. sonnei*, 615 *S. flexneri*). | *ISEcpl* carriage rate 90.09%; *ISEcpl*-CTX-M element rate 67.19%; higher in *blaCTX-M-9* group (95.59%) vs *blaCTX-M-1* group (91.95%); spacer lengths: 42,45,48,127 bp; *ISEcpl*+spacer+*blaCTX-M* significantly increased MICs for ceftriaxone, cefotaxime, cefuroxime, cephalothin, cefepime, ceftazidime. | | Moderate quality |
| Stefanovic et al. 2025 [7] | Canada (Vancouver), 2015–2022 | Retrospective observational study | 163 patients with *S. sonnei* infection (50 historical 2015–2020, 113 recent 2021–2022) | Cases increased from 8.3 to 56.5/year; population shifted from 98% MSM (historical) to 77% PEH (recent); severe shigellosis 14% to 61% (p<0.001); MDR to first-line agents + azithromycin 18% to 96%; dominant clone 3.6.1.1.2 (CipR.MSM5) with triple QRDR mutations and *pKSR100* plasmid. | | Moderate quality |
| Sharma et al. 2024 [8] | India, 2024 | Review | 20 studies | Cefotaxime has lower protein binding (35% vs 95%); ceftriaxone higher CDI/cholelithiasis risk | | Moderate quality |
| Zhao et al. 2024 [9] | China, 2024 | *In vitro* study | *Shigella dysenteriae* strain | Phage-antibiotic synergy with polymyxin B, sulfadiazine, TMP (>95% reduction) | | Moderate quality |
| Muzembo et al. 2023 [10] | South Asia, 2000–2022 | Review | 91 studies (384,323 stool samples) from Bangladesh, India, Nepal, Pakistan, Sri Lanka | Pooled *Shigella* prevalence 7% (95% CI: 6–7%); higher in children <5 years (10%), rural areas (12%), and PCR-based studies (15%). *S. flexneri* most common serogroup (58%), followed by *S. sonnei* (19%), *S. boydii* (10%), *S. dysenteriae* (9%). *S. flexneri* serotype 2a was dominant (36%). | | Moderate quality |
| Tansarli et al. 2023 [11] | USA (Seattle, WA), 2017–2022 | Observational study | 171 *Shigella* isolates (112 *S. flexneri*, 55 *S. sonnei*, 1 *S. boydii*) from adult patients | 51% MDR; 10 *S.* *flexneri*, 9 *S. sonnei* lineages; MSM→homeless spread; cipro R (lineage 3, *gyrA*/*parC*), azithromycin R (*pKSR100*); international links (China, UK, Switzerland); empirical therapy appropriate in 70%. | | Moderate quality |
| Matanza et al. 2023 [12] | Not applicable | Review | Not applicable | *S. sonnei* outcompetes *S. flexneri* with economic development; unique O-antigen, capsule, T6SS, colicins; less invasive; emerging XDR (*blaCTX-M*, mcr-1); no vaccine | | Low quality |
| Blacksell et al. 2023 [13] | Multi-country, 2023 | Review | Not applicable | LAIs underreported; infectious dose 10–200; insufficient evidence for disinfectant efficacy | | Low quality |
| Lefevre et al. 2023 [14] | France, 2005–2021 | Randomized trials | 7,121 *S. sonnei* isolates | First XDR *S. sonnei* detected in 2015; sharp increase to 22.3% (99/444) by 2021. XDR defined as resistance to CIP, AZM, and 3GCs. Predominant cluster X10 (genotype 3.6.1.1.2 CipR.MSM5, 102/164 XDR) associated with MSM networks. Resistance mechanisms: QRDR mutations (*gyrA*/*parC*) for CIP; *mph(A)*/erm(B) for AZM; diverse *blaCTX-M* genes (CTX-M-27, -15, -3, -55, -134) on *IncFII*, *IncI1*, *IncB/O/K/Z* plasmids or chromosome. | | Moderate quality |
| van den Beld et al. 2023 [15] | Netherlands, 2019–2021 | Observational study | 109 *Shigella* isolates (55 *S. sonnei*, 52 *S. flexneri*, 1 *S. boydii*, 1 *S. dysenteriae*) from Amsterdam region | | 49% of isolates in 14 clusters; 9/14 clusters MSM-associated; higher resistance in MSM: ciprofloxacin (89% vs 33%), azithromycin (58% vs 17%); international relatedness | Moderate quality |
| Yang et al. 2023 [16] | China (Beijing), 2023 | Review | Not applicable | | *Shigella* resistant to ciprofloxacin, azithromycin, ceftriaxone globally; MDR plasmids (*IncFII*, *IncI1*, *IncI2*, *IncB/O/K/Z*) carrying *blaCTX-M*, *mphA*, *ermB*, *qnr*, mcr; regional differences: high azithromycin/ceftriaxone resistance in Asia, high MDR among MSM in Europe/Americas. | Low quality |
| Jacqueline et al. 2023 [17] | Spain, 2021–2022 | Observational study | 37 *S. sonnei* isolates from 12 autonomous communities | | 33/37 genetically related to UK XDR outbreak (ST152, *IncFII*, *blaCTX-M-27*, *gyrA* S83L, *mphA*, *aadA5*, *sul1*, *dfrA1*); 4 additional genetically distant MDR strains (ST3075, ST5390). | Moderate quality |
| Choia et al. 2023 [18] | USA (Texas), 2023 | Case report | Immunocompromised adult male (HIV+, CD4 828) with acute diarrhea | | Isolate resistant to ciprofloxacin, azithromycin, ceftriaxone, TMP-SMX, ampicillin (all first-line agents); susceptible to fosfomycin, carbapenems, tigecycline. MALDI-TOF misidentified as *E. coli*; WGS confirmed *S. sonnei* ST152, carrying *blaCTX-M-27*, *gyrA* S83L, *qnrB19*, *mph(A)*, *sul1*, *sul2*, *dfrA1*, *dfrA17*. | Low quality |
| Coluccia et al. 2023 [19] | Italy, 2023 | *In vitro* study | *S. flexneri* M90T (wild-type) and its isogenic mutants | | Δ*acrAB* reduces intracellular survival and cell-to-cell spread; Δ*acrA* and Δ*acrB* (D408A) impair viability in epithelial cells; AcrD deletion has no effect; NMP treatment phenocopies AcrB defect. | Moderate quality |
| Moreno-Mingorance et al. 2023 [20] | Spain (Barcelona), 2020–2021 | Retrospective observational study | 37 *S. sonnei* and 43 *S. flexneri* isolates from 80 patients (50 MSM, median age 36.2 years) | | 68% of MSM-associated *S. sonnei* were XDR (*blaCTX-M-27*, resistant to azithromycin, ciprofloxacin, co-trimoxazole, cephalosporins); 14% of MSM-associated *S. flexneri* were MDR (*blaCTX-M-27*, fluoroquinolone-resistant); *S. sonnei* cluster belonged to CipR.MSM5 genotype (3.6.1.1.2) linked to UK outbreak; first emergence of ESBL-producing *S. flexneri* 2a among MSM. | Moderate quality |
| Ahamed et al. 2023 [21] | India, 2023 | *In vitro* study | 50 MDR clinical *Shigella* isolates | | Broad‑spectrum lytic activity against *S. flexneri*, *S. dysenteriae*, *S. sonnei* (not *S. boydii*). Stable at pH 3–11 and up to 50 °C. Latent period 5–20 min, burst size 71–265 PFU/cell. Genomes ~50.4 kb, no integrase/virulence genes. On raw chicken, phage cocktail reduced *Shigella* load by ~2 log10 within 72 h. Sfin‑2 receptor: LPS + protein; Sfin‑6: LPS only. | High quality |
| Mason et al. 2023 [22] | UK/Australia/Belgium/France/USA, 2023 | Observational study | 3,304 *S. sonnei* isolates from UK (2016–2021), Australia, Belgium, France, USA | | XDR *S. sonnei* outbreak strain (resistant to ciprofloxacin, azithromycin, ceftriaxone) belonging to CipR.MSM5 subclade; most recent common ancestor in 2018; carries low‑fitness‑cost *IncFII* plasmid *p893816*‑like with *blaCTX-M-27*, *mph(A)*, erm(B), *qnrB1*, tet(A), *dfrA17,* *aadA5*; predominantly in MSM (84% of UK outbreak isolates). Plasmid also found in *S. flexneri* 3a from Australia (2018). | Moderate quality |
| Haidar-Ahmad et al. 2023 [23] | Not applicable (review) | Review | Not applicable | | *Shigella* is an *E. coli* pathovar; virulence requires *pINV* plasmid encoding T3SS and IcsA; *pINV* maintenance differs between *S. flexneri* (*vapBC*, *gmvAT*, *ccdAB*, *parAB*, *stbAB*) and *S. sonnei* (*vapBC*, *relBE*); master regulators *VirF*, *VirB*, *MxiE* control T3SS expression; *H-NS* silences virulence at 30°C. | Moderate quality |
| Gaudreau et al. 2022 [24] | Canada (Montreal), 2017–2019 | Randomized trials | 32 men (31 MSM) with MDR *S. sonnei* infection | | All isolates resistant to ceftriaxone (*blaCTX-M-27*), azithromycin (*mphA*, *ermB*), nalidixic acid, TMP-SMX, tetracycline; reduced ciprofloxacin susceptibility (*gyrA* S83L); median 6 hqSNV differences; phylogenetic cluster (PD5000019750.208, genotype 3.7.29.1.4.1) linked to Australia, US, UK; 1 microbiological failure with ciprofloxacin. | High quality |
| Sriyapai et al. 2022 [25] | Thailand, 2022 | Observational study | 53 *Shigella* (cefotaxime-resistant) | | ESBL-positive rate 98.1%; *blaCTX-M-15* dominant; first report of *qnrS* and QRDR mutations in Thailand | Moderate quality |
| Sabour et al. 2022 [26] | Iran (Ardabil), 2019–2020 | Observational study | 1,280 fecal samples from children with diarrhea | | *Shigella* prevalence 3.8% (49/1,280): *S. sonnei* 85.7%, *S. flexneri* 10.2%, *S. dysenteriae* 4%; ESBL producers 10.2% (5/49); *blaCTX-M* 65.3%, *blaTEM* 61.2%, *blaSHV* 0%; ERIC-PCR: 42 *S. sonnei* into 6 clusters. | Moderate quality |
| Sen et al. 2022 [27] | Australia, 2022 | *In vitro* study | *S. flexneri* serotype 1c (wild-type, Δ*ynfA*, Δ*emrE*, Δ*ynfA*+Δ*emrE*, complemented strains, and site-directed YnfA mutants) | | YnfA is an SMR family efflux pump; deletion increases susceptibility to ethidium bromide, acriflavine, methyl viologen, crystal violet; reduces efflux activity; critical residues: E15A, G18A, Y60A impair function; EmrE has greater efflux capacity than YnfA; double knockout (Δ*ynfA*+Δ*emrE*) severely reduces transport. | High quality |
| Tinney et al. 2022 [28] | USA, 2022 | *In vitro* study | *S. flexneri* strains (wild-type, *ompA*⁻, *ompC*⁻, *ompA*⁻/C⁻) and *Shigella* phages *Sf22* and *KRT47* | | *OmpC* is necessary for *Sf22* and *KRT47* infection; resistance most commonly via *ompC* deletion or premature stop codons; point mutation N237T in *OmpC* reduced plating efficiency to <10⁻⁶; *OmpC* loops 5 and 7 important for phage recognition. | Moderate quality |
| MacLennan et al. 2022 [29] | Global, 2019 | Review | Estimated 148,202 deaths (all ages) from shigellosis, including 93,831 children <5 years | | Most advanced: bivalent *S. sonnei*/*S. flexneri* 2a conjugate vaccine (Phase 3 in China). Other candidates: ShigETEC (Phase 2), bioconjugate (Phase 2 in Kenya), GMMA (to be tested in Kenyan children), synthetic conjugate (Phase 1), Invasin complex. A 4-valent vaccine covering *S. flexneri* 2a, 3a, 6 and *S. sonnei* could cover ~75% of global strains. Serum O‑antigen IgG is a correlate of protection. | Low quality |
| Micoli et al. 2022 [30] | Taiwan, 2001 (published 2005) | Retrospective cohort study | 182 children from two elementary schools; 47 culture-positive for *S. sonnei* | | First report of plasmid-mediated CMY-2 AmpC β-lactamase in *S. sonnei* causing ceftriaxone resistance. 96.2% of isolates from school B nonsusceptible to ceftriaxone. 42.8% (9/21) still culture-positive after 5 days ampicillin; 2/9 developed ampicillin resistance during treatment. PFGE showed closely related patterns (clonal outbreak). All ceftriaxone-resistant isolates carried CMY-2 and SHV-5 genes on plasmids. | Moderate quality |
| Martin et al. 2022 [31] | Switzerland (2010), US (2015–2016), Kenya (2019–ongoing) | Retrospective study | Total >700 subjects (adults, children 2–5 years, infants 9 months) | | Flexyn2a: 10 μg PS ± alum; phase IIb CHIM showed vaccine efficacy 30–50% against any shigellosis, >70% against severe diarrhea; significant reduction in disease severity; anti-LPS serum IgG correlated with protection. S4V quadrivalent ongoing in Kenyan infants (NCT04056117). Monovalent *S. dysenteriae* bioconjugate safe and immunogenic in adults. | Low quality |
| Shad et al. 2021 [32] | Not applicable | Review | Not applicable | | *S. sonnei* is emerging globally, replacing *S. flexneri* in LMICs; encodes T6SS for competitive advantage; O-antigen inhibits internalization and inflammasome activation; plasmid-mediated resistance (*blaCTX-M*, *qnr*, *mcr-1*) widespread; fluoroquinolone-resistant lineage spread from South Asia; no licensed vaccine available. | Low quality |
| Chiu et al. 2021 [33] | Taiwan, 2020 | *In vitro* | 194 strains | | *blaCMY-2* regulated by *ISEcp1*-*tnpA*-*blc*-*sugE*; host-dependent resistance phenotype | Moderate quality |
| Ashida et al. 2021 [34] | Japan, 2021 | Review | Not applicable | | *Shigella* induces pyroptosis in macrophages, inhibits apoptosis/pyroptosis/necroptosis in epithelial cells | High quality |
| Muthuramalingam et al. 2021 [35] | USA, 2017 | Review | Not applicable | | T3SS structure (needle, basal body, sorting platform), assembly, therapeutic/vaccine targets | High quality |
| Chung The et al. 2021 [36] | Vietnam/UK et al. 2021 | Observational cohort study | 1,804 isolates (1980–2013) | | Newly defined *S. flexneri* Lineage 8; clonal replacement strongly associated with TMP resistance | High quality |
| Houpt et al. 2021 [37] | Bangladesh, 2019 | Observational cohort study | 149 *Shigella* culture-positive patients (children and adults) | | Decreased susceptibility to azithromycin (DSA) associated with persistent diarrhea at day 5 (31% vs 12%; RR 2.66), culture positivity at day 5/6 (35% vs 5%; RR 5.26), and higher hospitalization (58% vs 39%; RR 1.49). *S. sonnei* predominated among DSA (67%) and MDR strains (54%). MIC ≥16 μg/mL for *S. flexneri* predictive of poor outcome. | Moderate quality |
| Wong et al. 2021 [38] | Multi-country, 2021 | *In vitro* study | *E. coli* single cells (hundreds) | | Turgor pressure is main lysis driver; MSCs insufficient; hypoosmotic shock may enhance β-lactam killing | High quality |
| Stenhouse et al. 2021 [39] | Malawi, 2012–2015 | Observational study | 8 *Shigella* isolates (*S. flexneri* 3a, 4av, 6; *S. boydii* 2) from children <5 years hospitalized with acute gastroenteritis | | All *Shigella* isolates MDR; one *S. flexneri* 4av carried *qnrS1* (fluoroquinolone resistance); MDR plasmids shared across multiple serotypes and continents | Low quality |
| Yang et al. 2021 [40] | NF | *In vitro* study | 1 *Shigella boydii* strain | | *p2246-CTXM* carries erm(B) and *blaCTX-M-14* within a truncated *Tn6295* element. | High quality |
| Van den Bossche et al. 2021 [41] | Belgium, 2019 | Randomized trials | 47 symptomatic cases (37 children, 10 adults) in youth camp 1; 20 ill children in camp 2; total 9 isolates sequenced (7 from camp 1, 1 from camp 2, 1 from lab technician) | | All sequenced isolates were *S. sonnei* with 0 SNP differences; strain belonged to Holt lineage III; closest international match (1 allele difference) was a 2019 UK isolate; travel history to Central America (Mexico, Costa Rica, Curaçao) among UK/Netherlands isolates in same cluster. | High quality |
| Shahin et al. 2020 [42] | Global, 2019 (data)/2022 (publication) | Review | Global estimates: 148,202 deaths (all ages) from shigellosis in 2019; 93,831 in children <5 years | | Most advanced: bivalent *S. sonnei*/*S. flexneri* 2a conjugate vaccine (Phase 3 in China). A 4‑valent vaccine covering *S. flexneri* 2a, 3a, 6 and *S. sonnei* could cover ~75% of global strains. Serum O‑antigen IgG is a correlate of protection. No licensed vaccine after >100 years. | Low quality |
| Collins et al. 2020 [43] | USA, 2020 | Case series | 4 patients (3 MSM, 2 HIV+) | | Treatment failure with oral third-gen cephalosporins despite ceftriaxone-susceptible isolates | Low quality |
| Spellerberg et al. 2020 [44] | Germany, 2020 | Case series | 3 patients | | ESBL emergence 6–49 days after cephalosporin therapy; horizontal gene transfer in gut | Low quality |
| Schnupf et al. 2019 [45] | Not applicable | Review | Not applicable | | Comprehensive overview of *Shigella* pathogenesis: T3SS structure and regulation, invasion mechanisms, macrophage pyroptosis, epithelial cell invasion and cytosolic replication, autophagy evasion, subversion of NF-κB and MAPK pathways, inhibition of adaptive immunity, and metabolic adaptation. | Moderate quality |
| Anderson et al. 2019 [46] | 79 LMICs, 2025–2034 | Cost-effectiveness modeling study | Children under 5 years in 79 low- and lower-middle-income countries | | Estimated 340,300 *Shigella* deaths over 10 years; vaccine could prevent 126,600 direct deaths and 34,200 stunting-associated deaths; ICERs range from 955  /DALY(EMRO)to 955/DALY(EMRO)to649,033/DALY (EURO); cost-effective in 29 countries (ICER <3×GDP). | Low quality |
| Ranjbar et al. 2019 [47] | Iran, 2019 | Review | Not applicable | | Resistance mechanisms (efflux pumps, ESBL, QRDR, plasmids); new therapies (nanoparticles, phages, vaccines) | Low quality |
| Anderson et al. 2019 [48] | USA, 2019 | Modeling study | 79 LMICs, children <5 years | | 196 million annual ETEC + *Shigella* diarrhea episodes; stunting increases indirect deaths by 24–28% | Low quality |
| Pasqua et al. 2019 [49] | Not specified, 2019 | *In vitro* experimental study | *S. flexneri* M90T strain; U937 macrophage-like cells and Caco-2 epithelial cells | | *emrKY* induced 32-fold in macrophages (not in epithelial cells); activation requires EvgS/EvgA two-component system in response to mild acidic pH (6.0) and high K⁺; Δ*emrKY* mutant shows reduced survival in macrophages (competitive index 0.67). | High quality |
| Kotloff et al. 2018 [50] | Not applicable (review) | Review | Not applicable | | *Shigella* causes ~188 million cases and 164,300 deaths annually; *S. flexneri* dominant in LMICs, *S. sonnei* in HICs; rising antimicrobial resistance (ciprofloxacin, azithromycin, ceftriaxone); MSM and travelers drive intercontinental spread; WHO recommends ciprofloxacin as first-line; vaccines in development. | Moderate quality |
| Williams et al. 2018 [51] | International, 2018 | Review | 9 studies (children and adults with shigellosis) | | WHO guidelines (ciprofloxacin first-line) remain supported; ciprofloxacin resistance in Asia-Africa increased to 29.1% (2007–2009) but clinical failure low; azithromycin appropriate second-line where ciprofloxacin resistance high; cefixime alternative but risk of ESBL spread; no superior class identified. | Moderate quality |
| Liu et al. 2018 [52] | China (Jiangsu), 2013–2015 | Observational study | 282 *S. flexneri* isolates from patients with bacillary dysentery | | 34.4% resistant to cefotaxime, 24.1% to ceftazidime; 75.3% of resistant isolates ESBL-positive; dominant subtypes: *CTX-M-14* (10.3% of all isolates) and CTX-M-55 (6.0%) | Moderate quality |
| Mattock et al. 2017 [53] | UK, 2017 | Review | Not applicable | | Spatiotemporal model of virulence factors (barrier disruption → inflammation → adhesion/entry → replication/spread → immune evasion) | Low quality |
| Brown et al. 2017 [54] | Australia, 2017 | Observational study | 160 isolates (2013–2014) | | Azithromycin susceptibility 86.9% (but 40% in *S. flexneri*); ciprofloxacin resistance 35%; propose species-specific azithromycin ECOFF | Moderate quality |
| Aminshahidi et al. 2017 [55] | Iran (Shiraz), 2014–2015 | Observational study | 41 *Shigella* isolates from children <18 years with acute diarrhea | | *Shigella* prevalence 37.3% (41/110 positive samples); 80.5% *S. flexneri*, 19.5% *S. sonnei*; ESBL production 53.7%; resistance rates: ampicillin 95.1%, TMP-SMX 73.2%, azithromycin 21.9%, ciprofloxacin 14.6%, cefotaxime 53.7%, ceftriaxone 56.1%; MDR phenotype 24.4% (resistant to CTX, CTR, CAZ, AMP, SXT, ATH). | Moderate quality |
| Parajuli et al. 2017 [56] | Nepal (Kathmandu), 2016 | Case report | 2 siblings (6-month-old female, 2.5-year-old male) with acute dysentery | | Isolates: *S. flexneri*, MDR (resistant to ampicillin, azithromycin, co-trimoxazole, ceftazidime, cefixime, ciprofloxacin, gentamicin); ESBL-positive (CTX-M type); clinical failure with ceftriaxone; recovered after piperacillin-tazobactam. | Low quality |
| Nüesch-Inderbinen et al. 2016 [57] | Switzerland, 2004–2014 | Observational study | 344 *Shigella* isolates (179 *S. sonnei*, 107 *S. flexneri*, 33 *S. dysenteriae*, 25 *S. boydii*) | | 78.5% MDR; ciprofloxacin resistance: 15% *S. sonnei*, 8.4% *S. flexneri*; ESBL (CTX-M) in 4.5% *S. sonnei*; *mph(A)* in 2% (2 *S. sonnei*, 5 *S. flexneri*); QRDR mutations (e.g., GyrA Ser83Leu + ParC Ser80Ile) linked to ciprofloxacin resistance. | Moderate quality |
| von Seidlein et al. 2016 [58] | Vietnam/UK et al. 2016 | Observational study | 60 ciprofloxacin-resistant *S. sonnei* + 133 global isolates | | All ciprofloxacin-resistant *S. sonnei* is single clone; three-step QRDR mutations (*gyrA*-S83L→*parC*-S80I→*gyrA*-D87G); South Asia origin | Moderate quality |
| Klontz et al. 2015 [59] | USA, 2015 | Review | Not applicable | | History of resistance (1940s–2015); current main drugs (ciprofloxacin, azithromycin, ceftriaxone) all threatened | Low quality |
| Rashid et al. 2015 [60] | Bangladesh, 2005 | *In vitro* study | 1 *S. sonnei* isolate from a shigellosis patient | | *S. sonnei* and co-isolated *E. coli* both resistant to ceftriaxone, cefotaxime, ceftazidime, cefixime; ESBL-positive; transferable 50 MDa plasmid conferred TGC resistance to *E. coli* K12 and *S. sonnei* recipients | Low quality |
| Li et al. 2015 [61] | China (Shanghai), 2007 | Observational study | 216 *Shigella* isolates (123 *S. flexneri*, 92 *S. sonnei*, 1 *S. dysenteriae*) | | ESBL prevalence 18.1% (39/216); all ESBL-positive carried *blaCTX-M* (CTX-M-14 69.2%, CTX-M-15 15.4%, CTX-M-3 10.3%, CTX-M-55 5.1%); all *blaCTX-M* transferable with *ISEcp1* upstream; *IncI1* (58.9%) and *IncFII* (23.1%) dominant replicons. | Moderate quality |
| Li et al. 2015 [62] | Not applicable (review) | Review | Not applicable | | In *S. flexneri*, efflux pumps (including AcrAB overproduction) contribute to high-level fluoroquinolone resistance; inactivation of MFS pump MdfA reduces norfloxacin MIC by 8-fold. | Moderate quality |
| Sjölund Karlsson et al. 2013 [63] | USA, 2013 | Randomized trials | 43 outbreak cases, 4 isolates submitted | | Azithromycin MIC >16 µg/mL; *mphA* on plasmid; first such outbreak in USA | High quality |
| Oommen et al. 2013 [64] | India (Kerala), 2013 | Case report | 1 child (3-year-old male) with dysentery | | Isolate: CTX-M-type ESBL and AmpC producer; resistant to ampicillin, co-trimoxazole, chloramphenicol, nalidixic acid, ciprofloxacin; ceftriaxone MIC ≥64 µg/mL; clinical failure with ceftriaxone, resolution with meropenem. | Low quality |
| Kim et al. 2012 [65] | UK, 2012 | Review | Not applicable | | Stx direct toxicity + complement activation + VEGF dysregulation; antibiotics increase HUS risk; eculizumab limited evidence | Moderate quality |
| Porter et al. 2012 [66] | International, 1946–2010 | Review | 19 studies (9 *Shigella* strains) in healthy adult volunteers (non-endemic regions) | | Diarrhea attack rates ≤75–80% for 2457T and 53G; dysentery rates stable (~40–56%); homologous protection 64–100%; higher attack rates with NaHCO₃ vs milk for 2457T. | Moderate quality |
| Taneja et al. 2012 [67] | India, 2012 | Observational study | 119 *S. flexneri* + 24 *S. dysenteriae* (2001–2009) | | 16.8% *S. flexneri* cephalosporin-resistant; *blaTEM* (100%), *blaCTX-M-15* (50%), *blaCMY-2* (35%) | Moderate quality |
| Jeon et al. 2012 [68] | Korea, 2012 | Case report | 1 adult female traveller returning from India | | *S. flexneri* resistant to ciprofloxacin (MIC 8 µg/mL), norfloxacin, ofloxacin, nalidixic acid; intermediate to levofloxacin; *gyrA* mutations (Ser83Leu, Asp87Asn) and *parC* (Ser80Ile); no PMQR genes; increased *tolC* expression (3.7-fold) with ciprofloxacin; clinical failure with ciprofloxacin, resolved with ceftriaxone. | Low quality |
| Christopher et al. 2010 [69] | India, 2010 (2019 confirmed no update) | Review | 16 RCTs, 1,748 participants | | Antibiotics shorten illness duration; insufficient evidence for superiority of any class; need local susceptibility data | High quality |
| Mandal et al. 2009 [70] | India, 2009 | Case report | 1 child (12 months) | | Ceftriaxone-resistant *S. flexneri*; cefoperazone-sulbactam effective | Low quality |
| Hrabák et al. 2008 [71] | Czech Republic, 2006 | Case report | 1 adult female traveller returning from China, Nepal, India | | *S. sonnei* isolate produced CTX-M-15 ESBL (pI 8.9); resistant to cefotaxime, ceftazidime, ampicillin, nalidixic acid, tetracycline, TMP-SMX; transferable *IncFII* plasmid carrying *blaCTX-M-15* with *ISEcp1* upstream. | Low quality |
| Nicolas et al. 2007 [72] | France, 2007 | Review | Not applicable | | Shigellosis (bacillary dysentery) caused by *S. sonnei* (predominant in industrialized countries, milder), *S. flexneri* (endemic in developing countries), *S. dysenteriae* (epidemic, severe, Shiga toxin). Transmission fecal-oral, low infectious dose. Multidrug resistance common. WHO recommends ciprofloxacin first-line; alternatives: azithromycin, ceftriaxone. No licensed vaccine. | Low quality |
| WHO 2005 [73] | Global, 2005 | Observational study | N/A (guideline for control of shigellosis, including *S. dysenteriae* type 1 epidemics) | | Ciprofloxacin 15 mg/kg twice daily for 3 days in children (500 mg twice daily in adults) is drug of choice. Ampicillin, co‑trimoxazole, nalidixic acid no longer recommended due to resistance. Treatment within 48 h improves outcomes. Case fatality up to 15% in hospitalized Sd1 patients. Preventive measures: hand‑washing, safe water, sanitation, breastfeeding, food safety. | Low quality |
| Hata et al. 2005 [74] | Japan, 2003 | *In vitro* study | 8 *S. flexneri* 2b isolates from a food poisoning outbreak (1 with additional fluoroquinolone resistance) | | Identified novel plasmid-mediated quinolone resistance gene *qnrS* (59% amino acid identity to Qnr); *pAH0376* (47 kb) conferred low-level resistance to nalidixic acid, ciprofloxacin, and cephaloridine; co-harbored *blaTEM-1* | Moderate quality |
| Huang et al. 2005 [75] | Taiwan, 2001 | Randomized trials | 47 children (21 from school A, 26 from school B) with *S. sonnei* infection | | 55.3% of isolates resistant to ampicillin; 96.2% of school B isolates ceftriaxone-nonsusceptible; PFGE showed closely related patterns; ceftriaxone resistance mediated by plasmid-borne CMY-2 AmpC β-lactamase (first report in *S. sonnei*); two isolates from school A acquired ampicillin resistance during treatment. | High quality |
| Rahman et al. 2004 [76] | Bangladesh, 2004 | Observational study | 160 isolates (2001–2002) | | 4 isolates ceftriaxone-resistant (2.5%); ESBL (CTX-M) and *AmpC*-like enzymes; plasmids transferable | Moderate quality |
| Tauxe et al. 1990 [77] | USA, 1985–1986 | Randomized trials | 270 *Shigella* isolates (252 with clinical data); all ages | | 32% ampicillin-R, 7% TMP-SMX-R, 0.4% nalidixic acid-R; 20% travel-associated; travel linked to TMP-SMX-R (20% vs 4%, RR=3.3) | Moderate quality |
| Kabir et al. 1986 [78] | Bangladesh, 1985–1986 | Randomized trials | 94 adult males with acute dysentery (52 *S. dysenteriae*, 38 *S. flexneri*, 4 other) | | Both ceftriaxone and ampicillin reduced fever duration and stool frequency on certain days vs placebo. Ampicillin shortened positive stool culture duration (1.1 vs 2.6 days, p<0.05); ceftriaxone did not. Ceftriaxone reduced cumulative stool frequency in *S. flexneri* (p<0.05) but not *S. dysenteriae*. | High quality |

1.	Shahi F, Abbasi Montazeri E, Khaghani S, Moradi M, Ahmad Khosravi N, Saki M. Investigation of the azithromycin resistance rate and related genes involved in this resistance in clinical *Shigella* species collected from children with diarrhea in Ahvaz, Southwest Iran. Mol Biol Rep 2026, 53(1):353. <https://doi.org/10.1007/s11033-025-11420-3>.

2.	Global antibiotic resistance surveillance report 2025. 2025. <https://www.who.int/publications/i/item/9789240116337>

3.	Sati H, Carrara E, Savoldi A, Hansen P, Garlasco J, Campagnaro E, et al. The WHO Bacterial Priority Pathogens List 2024: a prioritisation study to guide research, development, and public health strategies against antimicrobial resistance. Lancet Infect Dis 2025, 25(9):1033-1043. <https://doi.org/10.1016/S1473-3099(25)00118-5>.

4.	Somda NS, Nyarkoh R, Tankoano A, Bonkoungou OJI, Tetteh-Quarcoo PB, Donkor ES. Molecular epidemiology of extended-spectrum beta-lactamases and carbapenemases-producing *Shigella* in Africa: a systematic review and meta-analysis. BMC Infect Dis 2025, 25(1):81. <https://doi.org/10.1186/s12879-024-10266-7>.

5.	Motlhaping T, Ramatla T, Thekisoe O, Khasapane NG, Lekota KE. Genomic insights into *Shigella* species isolated from small ruminants and manure in the North West Province, South Africa. Appl Microbiol Biotechnol 2025, 109(1):238. <https://doi.org/10.1007/s00253-025-13632-w>.

6.	Zhang H, He S, Pang X, He X, Liu X, Qiu S, et al. Regulatory role of *ISEcp1* and spacer sequences in *bla*(CTX-M)-mediated cephalosporin resistance in *Shigella*. J Glob Antimicrob Resist 2025, 44:127-134. <https://doi.org/10.1016/j.jgar.2025.06.005>.

7.	Stefanovic A, Alam ME, Matic N, Larnder A, Ritchie G, Gowland L, et al. Increased Severity of Multidrug-Resistant *Shigella sonnei* Infections in People Experiencing Homelessness. Clin Infect Dis 2025, 80(2):339-346. <https://doi.org/10.1093/cid/ciae575>.

8.	Sharma B, Chalikwar R, Bhalerao S, Gondane AA, Pawar D, Sharma A. Cefotaxime Versus Ceftriaxone: A Comprehensive Comparative Review. Cureus 2024, 16(9):e69146. <https://doi.org/10.7759/cureus.69146>.

9.	Zhao J, Chen B, Wang W, Kang Y, Hu E, Zhang Y, et al. Synergistic efficacy of phage Henu10 with antibiotics against *Shigella dysenteriae* with insight into phage resistance and fitness trade-offs. Front Cell Infect Microbiol 2024, 14:1499325. <https://doi.org/10.3389/fcimb.2024.1499325>.

10.	Muzembo BA, Kitahara K, Mitra D, Ohno A, Khatiwada J, Dutta S, et al. Burden of *Shigella* in South Asia: a systematic review and meta-analysis. J Travel Med 2023, 30(1). <https://doi.org/10.1093/jtm/taac132>.

11.	Tansarli GS, Long DR, Waalkes A, Bourassa LA, Libby SJ, Penewit K, et al. Genomic reconstruction and directed interventions in a multidrug-resistant Shigellosis outbreak in Seattle, WA, USA: a genomic surveillance study. Lancet Infect Dis 2023, 23(6):740-750. <https://doi.org/10.1016/s1473-3099(22)00879-9>.

12.	Matanza XM, Clements A. Pathogenicity and virulence of *Shigella sonnei*: A highly drug-resistant pathogen of increasing prevalence. Virulence 2023, 14(1):2280838. <https://doi.org/10.1080/21505594.2023.2280838>.

13.	Blacksell SD, Dhawan S, Kusumoto M, Le KK, Davis BJ, Summermatter K, et al. The Biosafety Research Road Map: The Search for Evidence to Support Practices in the Laboratory-*Shigella* spp. Appl Biosaf 2023, 28(2):96-101. <https://doi.org/10.1089/apb.2022.0046>.

14.	Lefevre S, Njamkepo E, Feldman S, Ruckly C, Carle I, Lejay-Collin M, et al. Rapid emergence of extensively drug-resistant *Shigella sonnei* in France. Nat Commun 2023, 14(1):462. <https://doi.org/10.1038/s41467-023-36222-8>.

15.	van den Beld M, Pijnacker R, van Dam A, Bovée L, Kwa D, Linde I, et al. Whole-genome sequencing of *Shigella* for surveillance purposes shows (inter)national relatedness and multidrug resistance in isolates from men who have sex with men. Microb Genom 2023, 9(4). <https://doi.org/10.1099/mgen.0.000978>.

16.	Yang C, Xiang Y, Qiu S. Resistance in Enteric *Shigella* and nontyphoidal Salmonella: emerging concepts. Curr Opin Infect Dis 2023, 36(5):360-365. <https://doi.org/10.1097/qco.0000000000000960>.

17.	Jacqueline C, Carrascoso GR, Gutiérrez-Fernández J, Rangel TV, Goterris L, Valdes FV, et al. Genetic Characterization of Extensively Drug-Resistant *Shigella sonnei* Infections, Spain, 2021–2022. Emerg Infect Dis 2023, 29(11). <https://doi.org/10.3201/eid2911.221746>.

18.	Choia H, Navarathnaa DH, Harstona BL, Hwanga M, Coronaa B, San Juana MR, et al. Case of Extensively Drug-Resistant *Shigella sonnei* Infection, United States. Emerg Infect Dis 2023, 29(8). <https://doi.org/10.3201/eid2908.230411>.

19.	Coluccia M, Beranger A, Trirocco R, Fanelli G, Zanzi F, Colonna B, et al. Role of the MDR Efflux Pump AcrAB in Epithelial Cell Invasion by *Shigella flexneri*. Biomolecules 2023, 13(5). <https://doi.org/10.3390/biom13050823>.

20.	Moreno-Mingorance A, Mir-Cros A, Goterris L, Rodriguez-Garrido V, Sulleiro E, Barbera MJ, et al. Increasing trend of antimicrobial resistance in *Shigella* associated with MSM transmission in Barcelona, 2020-21: outbreak of XRD *Shigella sonnei* and dissemination of ESBL-producing *Shigella flexneri*. J Antimicrob Chemother 2023, 78(4):975-982. <https://doi.org/10.1093/jac/dkad031>.

21.	Ahamed SKT, Rai S, Guin C, Jameela RM, Dam S, Muthuirulandi Sethuvel DP, et al. Characterizations of novel broad-spectrum lytic bacteriophages Sfin-2 and Sfin-6 infecting MDR *Shigella* spp. with their application on raw chicken to reduce the *Shigella* load. Front Microbiol 2023, 14. <https://doi.org/10.3389/fmicb.2023.1240570>.

22.	Mason LCE, Greig DR, Cowley LA, Partridge SR, Martinez E, Blackwell GA, et al. The evolution and international spread of extensively drug resistant *Shigella sonnei*. Nat Commun 2023, 14(1):1983. <https://doi.org/10.1038/s41467-023-37672-w>.

23.	Haidar-Ahmad N, Manigat FO, Silué N, Pontier SM, Campbell-Valois F-X. A Tale about *Shigella*: Evolution, Plasmid, and Virulence. Microorganisms 2023, 11(7). <https://doi.org/10.3390/microorganisms11071709>.

24.	Gaudreau C, Bernaquez I, Pilon PA, Goyette A, Yared N, Bekal S. Clinical and Genomic Investigation of an International Ceftriaxone- and Azithromycin-Resistant *Shigella sonnei* Cluster among Men Who Have Sex with Men, Montreal, Canada 2017-2019. Microbiol Spectr 2022, 10(3):e0233721. <https://doi.org/10.1128/spectrum.02337-21>.

25.	Sriyapai T, Pulsrikarn C, Chansiri K, Sriyapai P. Molecular characterization of extended-spectrum cephalosporin and fluoroquinolone resistance genes in Salmonella and *Shigella* isolated from clinical specimens in Thailand. Heliyon 2022, 8(12). <https://doi.org/10.1016/j.heliyon.2022.e12383>.

26.	Sabour S, Teimourpour A, Mohammadshahi J, Peeridogaheh H, Teimourpour R, Azimi T, et al. Molecular detection and characterization of *Shigella* spp. harboring extended-spectrum beta-lactamase genes in children with diarrhea in northwest Iran. Mol Cell Pediatr 2022, 9(1):19. <https://doi.org/10.1186/s40348-022-00152-0>.

27.	Sen T, Verma NK. Functional Role of YnfA, an Efflux Transporter in Resistance to Antimicrobial Agents in *Shigella flexneri*. Antimicrob Agents Chemother 2022, 66(7):e0029322. <https://doi.org/10.1128/aac.00293-22>.

28.	Tinney KR, Dover JA, Doore SM, Parent KN. *Shigella* viruses *Sf22* and *KRT47* require outer membrane protein C for infection. Biochimica et Biophysica Acta (BBA). Biomembranes 2022, 1864(7). <https://doi.org/10.1016/j.bbamem.2022.183920>.

29.	MacLennan CA, Steele AD. Frontiers in *Shigella* Vaccine Development. Vaccines (Basel) 2022, 10(9). <https://doi.org/10.3390/vaccines10091536>.

30.	Micoli F, Nakakana UN, Berlanda Scorza F. Towards a Four-Component GMMA-Based Vaccine against *Shigella*. Vaccines (Basel) 2022, 10(2). <https://doi.org/10.3390/vaccines10020328>.

31.	Martin P, Alaimo C. The Ongoing Journey of a *Shigella* Bioconjugate Vaccine. Vaccines (Basel) 2022, 10(2). <https://doi.org/10.3390/vaccines10020212>.

32.	Shad AA, Shad WA. *Shigella sonnei*: virulence and antibiotic resistance. Arch Microbiol 2021, 203(1):45-58. <https://doi.org/10.1007/s00203-020-02034-3>.

33.	Chiu CH, Lee JJ, Wang MH, Chu C. Genetic analysis and plasmid-mediated *bla*(CMY-2) in Salmonella and *Shigella* and the Ceftriaxone Susceptibility regulated by the ISEcp-1 *tnpA*-*bla*(CMY-2)-*blc*-*sugE*. J Microbiol Immunol Infect 2021, 54(4):649-657. <https://doi.org/10.1016/j.jmii.2020.01.008>.

34.	Ashida H, Suzuki T, Sasakawa C. *Shigella* infection and host cell death: a double-edged sword for the host and pathogen survival. Curr Opin Microbiol 2021, 59:1-7. <https://doi.org/10.1016/j.mib.2020.07.007>.

35.	Muthuramalingam M, Whittier SK, Picking WL, Picking WD. The *Shigella* Type III Secretion System: An Overview from Top to Bottom. Microorganisms 2021, 9(2). <https://doi.org/10.3390/microorganisms9020451>.

36.	Chung The H, Bodhidatta L, Pham DT, Mason CJ, Ha Thanh T, Voong Vinh P, et al. Evolutionary histories and antimicrobial resistance in *Shigella flexneri* and *Shigella sonnei* in Southeast Asia. Commun Biol 2021, 4(1):353. <https://doi.org/10.1038/s42003-021-01905-9>.

37.	Houpt ER, Ferdous T, Ara R, Ibrahim M, Alam MM, Kabir M, et al. Clinical Outcomes of Drug-resistant Shigellosis Treated With Azithromycin in Bangladesh. Clin Infect Dis 2021, 72(10):1793-1798. <https://doi.org/10.1093/cid/ciaa363>.

38.	Wong F, Wilson S, Helbig R, Hegde S, Aftenieva O, Zheng H, et al. Understanding Beta-Lactam-Induced Lysis at the Single-Cell Level. Front Microbiol 2021, 12:712007. <https://doi.org/10.3389/fmicb.2021.712007>.

39.	Stenhouse GE, Jere KC, Peno C, Bengtsson RJ, Chinyama E, Mandolo J, et al. Whole genome sequence analysis of *Shigella* from Malawi identifies fluoroquinolone resistance. Microb Genom 2021, 7(5). <https://doi.org/10.1099/mgen.0.000532>.

40.	Yang X, Liu X, Yang C, Chan EW-C, Zhang R, Chen S, et al. A Conjugative *IncI1* Plasmid Carrying erm (B) and *blaCTX-M-104* That Mediates Resistance to Azithromycin and Cephalosporins. Microbiol Spectr 2021, 9(2). <https://doi.org/10.1128/Spectrum.00286-21>.

41.	Van den Bossche A, Ceyssens P-J, Denayer S, Hammami N, van den Beld M, Dallman TJ, et al. Outbreak of Central American born *Shigella sonnei* in two youth camps in Belgium in the summer of 2019. Eur J Clin Microbiol Infect Dis 2021, 40(7):1573-1577. <https://doi.org/10.1007/s10096-021-04164-y>.

42.	Shahin K, Bouzari M, Komijani M, Wang R. A New Phage Cocktail Against Multidrug, ESBL-Producer Isolates of *Shigella sonnei* and *Shigella flexneri* with Highly Efficient Bacteriolytic Activity. Microb Drug Resist 2020, 26(7):831-841. <https://doi.org/10.1089/mdr.2019.0235>.

43.	Collins JP, Friedman CR, Birhane MG, Karp BE, Osinski A, Montgomery MW, et al. Evidence of Failure of Oral Third-Generation Cephalosporin Treatment for *Shigella sonnei* Infection. Open Forum Infect Dis 2020, 7(4):ofaa113. <https://doi.org/10.1093/ofid/ofaa113>.

44.	Spellerberg B, Rabsch W, Pietsch M, Denzer C, Posovszky C, Essig A, et al. Extended-spectrum beta-Lactamase Acquisition in Patients Receiving Systemic Cephalosporin Treatment for Salmonella spp. and *Shigella* spp. Infection. Clin Infect Dis 2020, 70(4):714-716. <https://doi.org/10.1093/cid/ciz468>.

45.	Schnupf P, Sansonetti PJ. *Shigella* Pathogenesis: New Insights through Advanced Methodologies. Microbiol Spectr 2019, 7(2). <https://doi.org/10.1128/microbiolspec.BAI-0023-2019>.

46.	Anderson JDt, Bagamian KH, Muhib F, Baral R, Laytner LA, Amaya M, et al. Potential impact and cost-effectiveness of future ETEC and *Shigella* vaccines in 79 low- and lower middle-income countries. Vaccine X 2019, 2:100024. <https://doi.org/10.1016/j.jvacx.2019.100024>.

47.	Ranjbar R, Farahani A. *Shigella*: Antibiotic-Resistance Mechanisms And New Horizons For Treatment. Infect Drug Resist 2019, 12:3137-3167. <https://doi.org/10.2147/IDR.S219755>.

48.	Anderson JD, Bagamian KH, Muhib F, Amaya MP, Laytner LA, Wierzba T, et al. Burden of enterotoxigenic *Escherichia coli* and *shigella* non-fatal diarrhoeal infections in 79 low-income and lower middle-income countries: a modelling analysis. Lancet Glob Health 2019, 7(3):e321-e330. <https://doi.org/10.1016/s2214-109x(18)30483-2>.

49.	Pasqua M, Grossi M, Scinicariello S, Aussel L, Barras F, Colonna B, et al. The MFS efflux pump EmrKY contributes to the survival of *Shigella* within macrophages. Sci Rep 2019, 9(1). <https://doi.org/10.1038/s41598-019-39749-3>.

50.	Kotloff KL, Riddle MS, Platts-Mills JA, Pavlinac P, Zaidi AKM. Shigellosis. Lancet 2018, 391(10122):801-812. <https://doi.org/10.1016/S0140-6736(17)33296-8>.

51.	Williams PCM, Berkley JA. Guidelines for the treatment of dysentery (shigellosis): a systematic review of the evidence. Paediatr Int Child Health 2018, 38(sup1):S50-s65. <https://doi.org/10.1080/20469047.2017.1409454>.

52.	Liu G, Qian H, Tang B, Chen Y, Kang H, Jiang F, et al. Prevalence and characterisation of third-generation cephalosporin-resistant *Shigella flexneri* isolates from Jiangsu Province, China, 2013-2015. J Glob Antimicrob Resist 2018, 15:283-287. <https://doi.org/10.1016/j.jgar.2018.08.012>.

53.	Mattock E, Blocker AJ. How Do the Virulence Factors of *Shigella* Work Together to Cause Disease? Front Cell Infect Microbiol 2017, 7:64. <https://doi.org/10.3389/fcimb.2017.00064>.

54.	Brown JD, Willcox SJ, Franklin N, Hazelton B, Howard P, Reinten T, et al. *Shigella* species epidemiology and antimicrobial susceptibility: the implications of emerging azithromycin resistance for guiding treatment, guidelines and breakpoints. J Antimicrob Chemother 2017, 72(11):3181-3186. <https://doi.org/10.1093/jac/dkx268>.

55.	Aminshahidi M, Arastehfar A, Pouladfar G, Arman E, Fani F. Diarrheagenic *Escherichia coli* and *Shigella* with High Rate of Extended-Spectrum Beta-Lactamase Production: Two Predominant Etiological Agents of Acute Diarrhea in Shiraz, Iran. Microb Drug Resist 2017, 23(8):1037-1044. <https://doi.org/10.1089/mdr.2017.0204>.

56.	Parajuli NP, Joshi G, Pardhe BD, Shakya J, Bhetwal A, Shakya S, et al. Shigellosis Caused by CTX-M Type ESBL Producing *Shigella flexneri* in Two Siblings of Rural Nepal: First Case Report from the Country. Case Rep Infect Dis 2017, 2017:1-5. <https://doi.org/10.1155/2017/1862320>.

57.	Nuesch-Inderbinen M, Heini N, Zurfluh K, Althaus D, Hachler H, Stephan R. *Shigella* Antimicrobial Drug Resistance Mechanisms, 2004-2014. Emerg Infect Dis 2016, 22(6):1083-1085. <https://doi.org/10.3201/eid2206.152088>.

58.	von Seidlein L, Chung The H, Rabaa MA, Pham Thanh D, De Lappe N, Cormican M, et al. South Asia as a Reservoir for the Global Spread of Ciprofloxacin-Resistant *Shigella sonnei*: A Cross-Sectional Study. PLOS Medicine 2016, 13(8). <https://doi.org/10.1371/journal.pmed.1002055>.

59.	Klontz KC, Singh N. Treatment of drug-resistant *Shigella* infections. Expert Rev Anti Infect Ther 2015, 13(1):69-80. <https://doi.org/10.1586/14787210.2015.983902>.

60.	Rashid H, Rahman M. Possible transfer of plasmid mediated third generation cephalosporin resistance between *Escherichia coli* and *Shigella sonnei* in the human gut. Infect Genet Evol 2015, 30:15-18. <https://doi.org/10.1016/j.meegid.2014.11.023>.

61.	Li J, Li B, Ni Y, Sun J. Molecular characterization of the extended-spectrum beta-lactamase (ESBL)-producing *Shigella* spp. in Shanghai. Eur J Clin Microbiol Infect Dis 2015, 34(3):447-451. <https://doi.org/10.1007/s10096-014-2244-2>.

62.	Li XZ, Plesiat P, Nikaido H. The challenge of efflux-mediated antibiotic resistance in Gram-negative bacteria. Clin Microbiol Rev 2015, 28(2):337-418. <https://doi.org/10.1128/CMR.00117-14>.

63.	Sjölund Karlsson M, Bowen A, Reporter R, Folster JP, Grass JE, Howie RL, et al. Outbreak of Infections Caused by *Shigella sonnei* with Reduced Susceptibility to Azithromycin in the United States. Antimicrob Agents Chemother 2013, 57(3):1559-1560. <https://doi.org/10.1128/aac.02360-12>.

64.	Oommen S, Pillai PM, Sushamabai S, Paul PJ. Cefotaximase and AmpC-producing *Shigella flexneri* in case of dysentery from southern India. Indian J Med Microbiol 2013, 31(1):77-79. <https://doi.org/10.4103/0255-0857.108734>.

65.	Kim JJ, Keir, Marks. Shigatoxin-associated hemolytic uremic syndrome: current molecular mechanisms and future therapies. Drug Des Devel Ther 2012. <https://doi.org/10.2147/dddt.S25757>.

66.	Porter CK, Thura N, Ranallo RT, Riddle MS. The *Shigella* human challenge model. Epidemiol Infect 2012, 141(2):223-232. <https://doi.org/10.1017/s0950268812001677>.

67.	Taneja N, Mewara A, Kumar A, Verma G, Sharma M. Cephalosporin-resistant *Shigella flexneri* over 9 years (2001-09) in India. J Antimicrob Chemother 2012, 67(6):1347-1353. <https://doi.org/10.1093/jac/dks061>.

68.	Jeon YL, Nam YS, Lim G, Cho SY, Kim YT, Jang JH, et al. Quinolone-resistant *Shigella flexneri* isolated in a patient who travelled to India. Ann Lab Med 2012, 32(5):366-369. <https://doi.org/10.3343/alm.2012.32.5.366>.

69.	Christopher PR, David KV, John SM, Sankarapandian V. Antibiotic therapy for *Shigella* dysentery. Cochrane Database Syst Rev 2010, 2010(8):CD006784. <https://doi.org/10.1002/14651858.CD006784.pub4>.

70.	Mandal J, Mondal N, Mahadevan S, Parija SC. Emergence of Resistance to Third-Generation Cephalosporin in *Shigella*--A Case Report. J Trop Pediatr 2009, 56(4):278-279. <https://doi.org/10.1093/tropej/fmp118>.

71.	Hrabák J, Empel J, Gniadkowski M, Halbhuber Zk, Rébl K, Urbášková P. CTX-M-15-Producing *Shigella sonnei* Strain from a Czech Patient Who Traveled in Asia. J Clin Microbiol 2008, 46(6):2147-2148. <https://doi.org/10.1128/jcm.00427-08>.

72.	Nicolas X, Granier H, Le Guen P. Shigellosis or bacillary dysentery. Presse Med 2007, 36(11 Pt 2):1606-1618. <https://doi.org/10.1016/j.lpm.2007.03.001>.

73.	Guidelines for the control of shigellosis, including epidemics due to *Shigella dysenteriae* type 1. <https://www.who.int/publications/i/item/9241592330>

74.	Hata M, Suzuki M, Matsumoto M, Takahashi M, Sato K, Ibe S, et al. Cloning of a Novel Gene for Quinolone Resistance from a Transferable Plasmid in *Shigella flexneri* 2b. Antimicrob Agents Chemother 2005, 49(2):801-803. <https://doi.org/10.1128/aac.49.2.801-803.2005>.

75.	Huang IF, Chiu C-H, Wang M-H, Wu C-Y, Hsieh K-S, Chiou CC. Outbreak of Dysentery Associated with Ceftriaxone-Resistant *Shigella sonnei* : First Report of Plasmid-Mediated CMY-2-Type AmpC β-Lactamase Resistance in S. sonnei. J Clin Microbiol 2005, 43(6):2608-2612. <https://doi.org/10.1128/jcm.43.6.2608-2612.2005>.

76.	Rahman M, Shoma S, Rashid H, Siddique AK, Nair GB, Sack DA. Extended-spectrum β-lactamase-mediated third-generation cephalosporin resistance in *Shigella* isolates in Bangladesh. J Antimicrob Chemother 2004, 54(4):846-847. <https://doi.org/10.1093/jac/dkh413>.

77.	Tauxe RV, Puhr ND, Wells JG, Hargrett-Bean N, Blake PA. Antimicrobial resistance of *Shigella* isolates in the USA: the importance of international travelers. J Infect Dis 1990, 162(5):1107-1111. <https://doi.org/10.1093/infdis/162.5.1107>.

78.	Kabir I, Butler T, Khanam A. Comparative efficacies of single intravenous doses of ceftriaxone and ampicillin for shigellosis in a placebo-controlled trial. Antimicrob Agents Chemother 1986, 29(4):645-648. <https://doi.org/10.1128/AAC.29.4.645>.
